# Anatomically modern human in the Châtelperronian hominin collection from the Grotte du Renne (Arcy-sur-Cure, Northeast France)

**DOI:** 10.1038/s41598-023-39767-2

**Published:** 2023-08-04

**Authors:** Arthur Gicqueau, Alexandra Schuh, Juliette Henrion, Bence Viola, Caroline Partiot, Mark Guillon, Liubov Golovanova, Vladimir Doronichev, Philipp Gunz, Jean-Jacques Hublin, Bruno Maureille

**Affiliations:** 1 Univ. de Toulouse Jean Jaurès, CNRS, Ministère de La Culture, TRACES, UMR5608 CNRS, F-31058 Toulouse, France; 2 Univ. Bordeaux, CNRS, Ministère de la Culture, PACEA, UMR5199, F-33600 Pessac, France; 3 Department of Human Evolution, Max Planck Institute for Evolutionnary Anthropology, Deutscher Platz 6, D-04103 Leipzig, Germany; 4 Department of Human Origins, Max Planck Institute for Evolutionary Anthropology, Deutscher Platz 6, D-04103 Leipzig, Germany; 5 Department of Anthropology, University of Toronto, Toronto, Canada; 6 Austrian Archaeological Institute (OeAI) at the Austrian Academy of Sciences (OeAW), Franz Klein-Gasse 1, 1190 Wien/Vienna, Austria; 7 Museum national d’histoire naturelle, Eco-Anthropologie, UMR7206, F-Paris, France; 8 Inrap, Boulevard de Verdun, F-76120 Le Grand Quevilly, France; 9 Laboratory of Prehistory, St. Petersburg, 199034 Russia; 10 Chaire Internationale de Paléoanthropologie, Collège de France, F-75231 Paris, France

**Keywords:** Evolution, Anthropology, Archaeology, Taxonomy

## Abstract

Around 42,000 years ago, anatomically modern humans appeared in Western Europe to the detriment of indigenous Neanderthal groups. It is during this period that new techno-cultural complexes appear, such as the Châtelperronian that extends from northern Spain to the Paris Basin. The Grotte du Renne (Arcy-sur-Cure) is a key site for discussing the biological identity of its makers. This deposit has yielded several Neanderthal human remains in its Châtelperronian levels. However, the last inventory of the paleoanthropological collection attributed to this techno-complex allowed the identification of an ilium belonging to a neonate (AR-63) whose morphology required a thorough analysis to assess its taxonomic attribution. Using geometric morphometrics, we quantified its morphology and compared it to that of 2 Neanderthals and 32 recent individuals deceased during the perinatal period to explore their morphological variation. Our results indicate a morphological distinction between the ilia of Neanderthals and anatomically modern neonates. Although AR-63 is slightly outside recent variability, it clearly differs from the Neanderthals. We propose that this is due to its belonging to an early modern human lineage whose morphology differs slightly from present-day humans. We also explore different hypotheses about the presence of this anatomically modern neonate ilium among Neanderthal remains.

## Introduction

**Figure 1:**
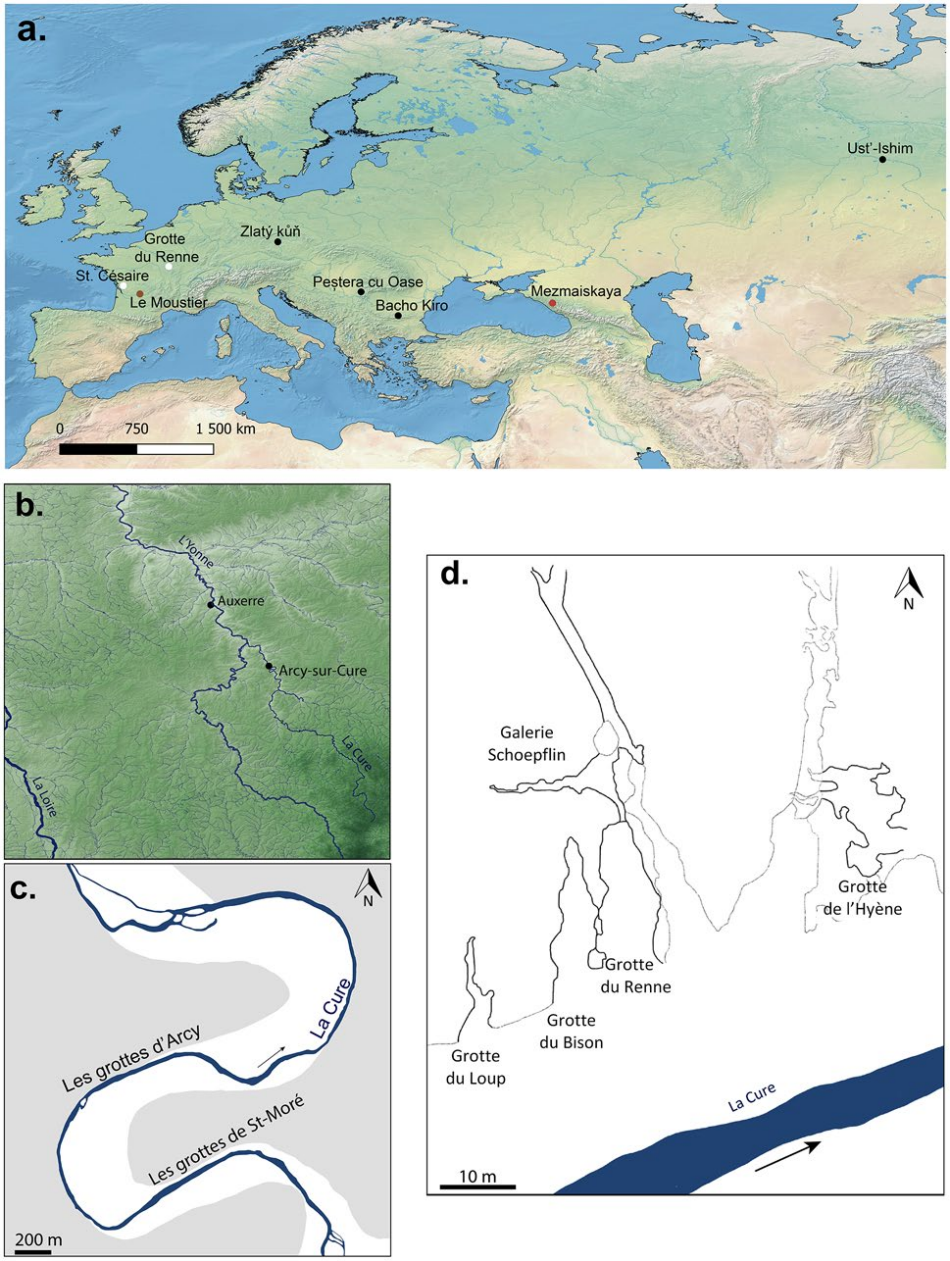
(**a**) Geographical distribution of sites with Neanderthal fossils discovered in Châtelperronian layers (white dots) and directly dated early anatomically modern humans predating 37 kya cal BP (black dots). The Mousterian sites of Le Moustier and Mezmaiskaya where the two perinatal Neanderthal ilia used as comparative sample in this study have been discovered are indicated by the red dots. The map was created by AG with QGIS software (version 3.22.5) http://www.qgis.org using Eurostat-Gisco vector, Natural Earth, Open Street Map data and Adobe Illustrator (version 4.1.2) https://www.adobe.com/products/illustrator.html. (**b**) Left, location of the site of Arcy-sur-Cure. The map was created by AG with QGIS software (version 3.22.5) http://www.qgis.org using Shuttle Radar Topography Mission, IGN vector and Open Street Map data and Adobe Illustrator (version 4.1.2) https://www.adobe.com/products/illustrator.html. (**c**) Location of the caves of Arcy-sur-Cure. Modified and drawn by JH, from David et al. (2006, 2009). (**d**) Map of the caves of Arcy-sur-Cure. Modified and drawn by JH, from Rocca et al. (2017).

The transition between the end of the Middle Paleolithic (MP) and the beginning of the Upper Paleolithic (UP) coincides with the decline of the Neanderthals and the expansion of the first anatomically modern human (AMH) groups in Western Europe. It was during this period, around 42,000 years ago (kya cal BP), that new techno-cultural complexes appeared in this area, discovered at the interface of underlying MP and overlying UP occupations. This episode of human plurality therefore raises questions about the biological identity of the bearers of these techno-complexes. Among these, the Châtelperronian, present in the north of Spain, the south-west, the center of France and in the Paris Basin between around 43,760 and 39,220 BP^1^, is the subject of a passionate scientific debate about the identity of its makers, Neanderthals or *Homo sapiens*, and the modalities of its emergence^2–25^. Recently, Gravina et al.^23^ conducted a taphonomic, spatial, and typo-technological reassessment of Châtelperronian lithic material from the *EJOP sup* level of La Roche-à-Pierrot (Saint-Césaire, Southwest France) (Fig. 1a) in which a well-preserved Neanderthal skeleton was uncovered^3^. The study showed that it is no longer possible to associate the Neanderthal human remains with Châtelperronian material given that the level in question contains “*an extremely limited quantity of Châtelperronian cultural material clearly mixed with an overwhelmingly Middle Palaeolithic component”* (Ref.^23^ p. 8). In the context of this debate, the Grotte du Renne (GDR) of Arcy-sur-Cure (Yonne, France)^2,7,12–15,17,24,26,27^, located in the Yonne Basin, 35 km south of Auxerre (France) (Fig. 1a and b), is now the only site where several Châtelperronian layers have yielded human remains, until now all assigned to Neanderthals^2,13,17,22,28,29^.

**Figure 2:**
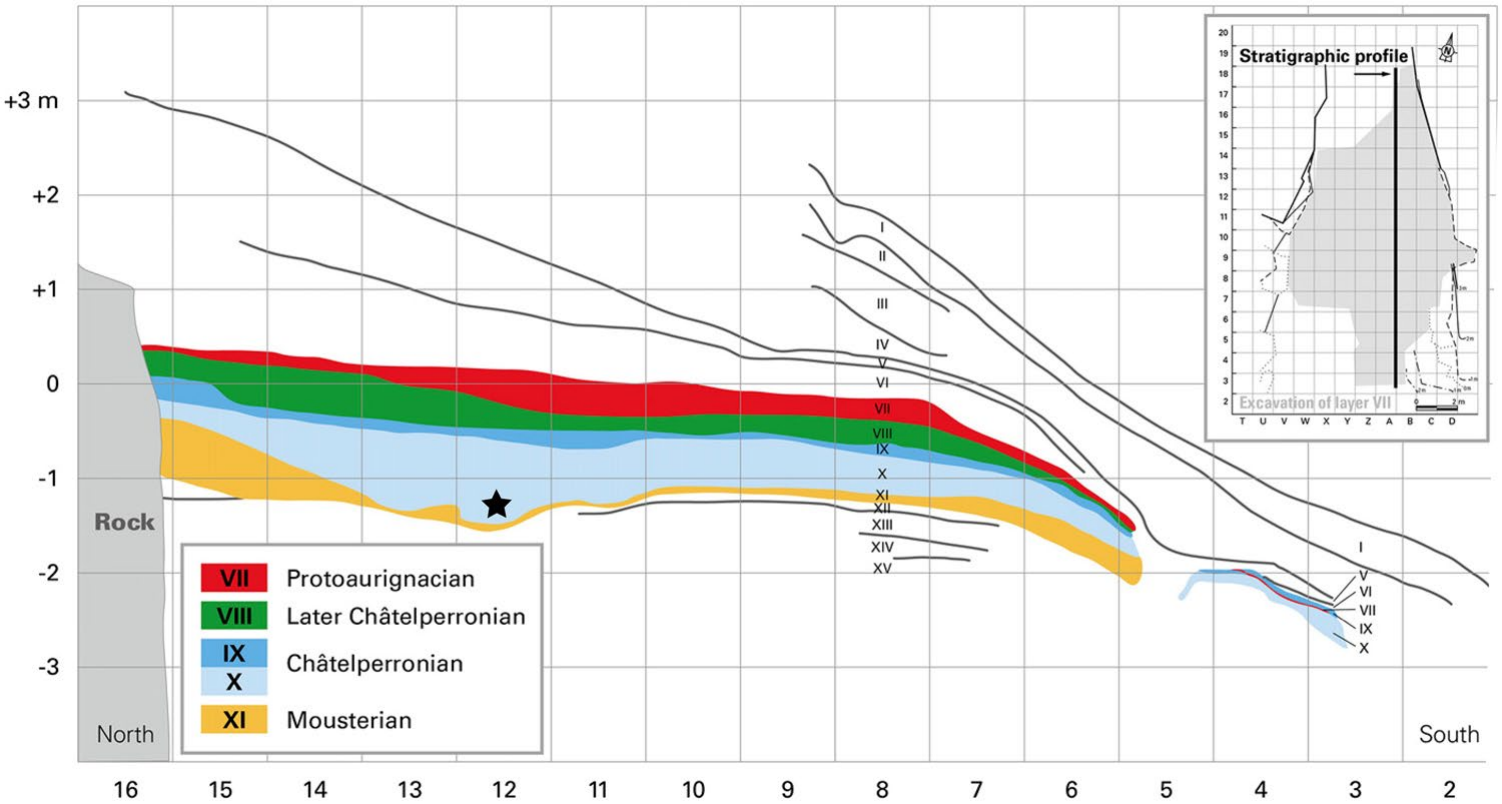
North–south stratigraphic section between the A and B raws of the excavation grid. Each square represents 1 m. The black star represents the projection of the localization of the perinatal ilium AR-63 discovered in the Xb2 sublayers. Modified from Hublin et al. (2012); original field drawing R. Humbert, archives A. Leroi-Gourhan (Connet, 2002).

Located at the opening of a karstic gallery oriented to the south on the left bank of the Cure river (Fig. 1c and d), the archaeological deposits of the GDR were excavated between 1949 and 1963 by A. Leroi-Gourhan. He identified 14 stratigraphic units, of which the eight lower ones (number VII to XIV from top to bottom) were described sub-horizontal in the cavity (Ref.^30^, Figure 1 p. 427 and Fig. 2). He recognized two Châtelperronian layers (X, IX) and one that he attributed to the Final Châtelperronian (VIII). The latter, situated between an underlying Mousterian layer (XI) and an overlying layer attributed to the Aurignacian (VII), yielded a rich assemblage of bone industry, ornaments^15,16,21,24,31,32^ and laminar lithic pieces^26,33–37^ associated with human remains identified as Neanderthals^2,13,17,22,29^.

According to some authors^10,19,20,38–41^, the discovery of these Neanderthal fossils in layers containing a lot of material with features considered as "typical" of the UP (production of blades, bone industries, ornaments and coloring materials) is a result of inter-stratigraphic disturbances that mixed Mousterian human remains with remains from overlying levels reported to the UP.

Based on field notes and surveys from the excavation notebooks of A. Leroi-Gourhan's team, a taphonomic analysis of different archaeological assemblages of the GDR was conducted for a first assessment of their integrity^42^. Although the results of this study show the existence of post-depositional processes that displaced remains in certain sectors, notably through the identification of refitting between some pieces from distinct subsets^43,44^ or the detection of runoff, it was emphasized that the movements occurred within the subsets of the same layer. Except in small localized areas, these indications of post-depositional movements do not call into question the major stratigraphic subdivisions initially defined and therefore the association of Châtelperronian material with layers X, IX and VIII.

To assess the age of the archaeological levels, seven radiocarbon dating projects have been conducted^14,20,36,45–48^. A recent synthesis of these results, carried out by Banks and d’Errico^49^ proposed a new chronology for the GDR archaeological sequence using OxCal 4.2^50^ and the IntCal13 calibration curve^51^. By combining the results of the two most recent studies, obtained by Accelerator Mass Spectometry (AMS) from remains from A. Leroi-Gourhan's excavations, and by employing a new probabilistic model based on all of the previous dating, Banks and d’Errico propose a chronological interval in agreement with the results of Hublin et al.^14^ regarding the Mousterian of layers XII and XI, i.e., between about 46 and 44.5 kya cal BP, and the early phase of the Châtelperronian, i.e., layers X and IX, which would have begun around 44.5 kya cal BP. However, the authors point out some differences between their model and that of Hublin et al. regarding the transition between the Châtelperronian and early Aurignacian: 39.5 kya cal BP for Banks and d’Errico and 41 kya cal BP for Hublin et al. Note that Higham et al.^52^ assign an age to the Châtelperronian occupations similar to that obtained by Hublin et al*.* The interpretations of these results stay questionable taking into account the analysis questioning the chronocultural integrity of the dated material^53^.

Concerning the age of the human remains unearthed in the Châtelperronian levels, only one piece (a cranial vault fragment from layer X, AR-14; Ref.^22^) assigned to the Neanderthal lineage on the basis of its mitochondrial DNA, has been dated and provided an age between 40,680 and 42,335 cal BP (2σ, 95.4%)^22,54^.

To date, studies of the human fossils of the GDR identified 64 remains attributed to layers X, IX and VIII. Among these 64 remains, 38 of them exhibit morphological and genetic traits assigning them to the Neanderthal lineage^2,13,17,22,28,29^. From a morphological point of view, this attribution is essentially based on the identification of dental features frequently observed in Neanderthals^2,17,28,29^. In addition, the architecture of the semicircular canals of the inner ear of a left temporal bone of an immature individual (Arcy 63 R C7 Xb 1544) showed clear Neanderthal affinities^13^. More recently, two cranial bone fragments (AR-14 and AR-30), first identified by ZooMs (Zooarchaeology by Mass Spectrometry), have been shown to carry Neanderthal-like mitochondrial DNA^22^.

**Figure 3:**
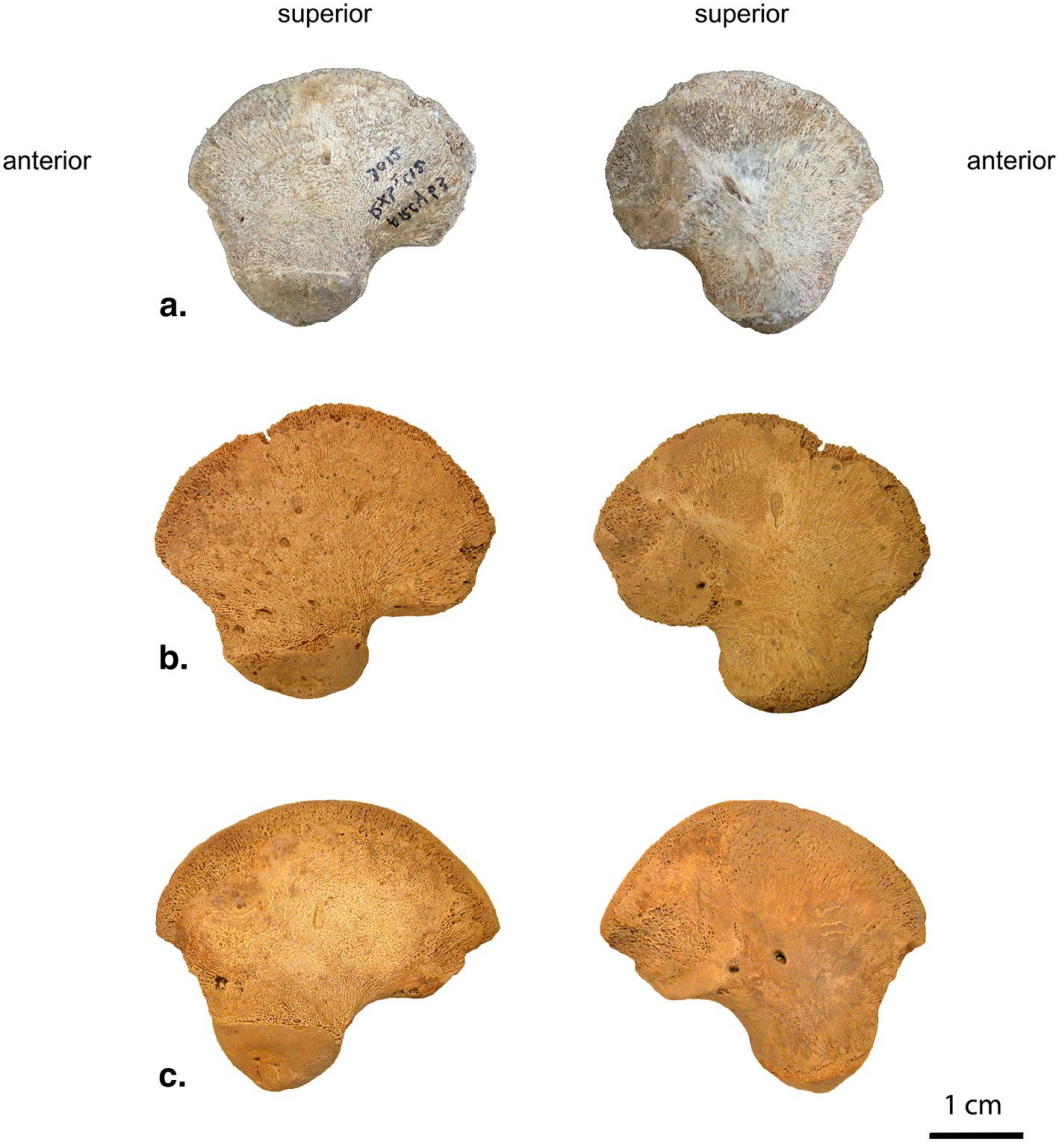
Lateral (left) and medial (right) view of (**a**) the right ilium (mirrored in left) of AR-63, (**b**) the left ilium of the perinatal Neanderthal LM2 and (**c**) the left ilium of a recent perinatal individual (T41).

In 2019, the paleoanthropological collection was reassessed and has been the subject of a new inventory^29^ leading to the identification of new human pieces. While all were previously reported to fall within the Neanderthal range of variation, a right ilium (Arcy 63 R Xb2 C12 3012; from here on AR-63, Fig. 3a) belonging to an individual deceased during the perinatal period given its shape and size^55–57^ has a morphology that required further study to assess its taxonomic affinities. It was possible to see that its shape was different from that of known Neanderthal neonate ilia^29,58–66^. However, AR-63 comes from the base of the Châtelperronian sequence of the GDR (Fig. 2), i.e. from level Xb2 in which 11 Neanderthal human remains have been identified^17^. By comparing AR-63 to the ilium of the Neanderthal neonate Le Moustier 2 (LM2) and to that of a recent neonate from Classic Kerma (T41, not T47 as written in Ref.^29,67^) it was clear that the morphology of AR-63, for some traits, is more similar to T41 than to LM2 (Fig. 3). It was therefore necessary to explore the morphological variability of recent neonate ilia and compare it to Neanderthal fossils and the ilium of AR-63 to discuss the taxonomic attribution of the latter.

## Results

**Figure 4:**
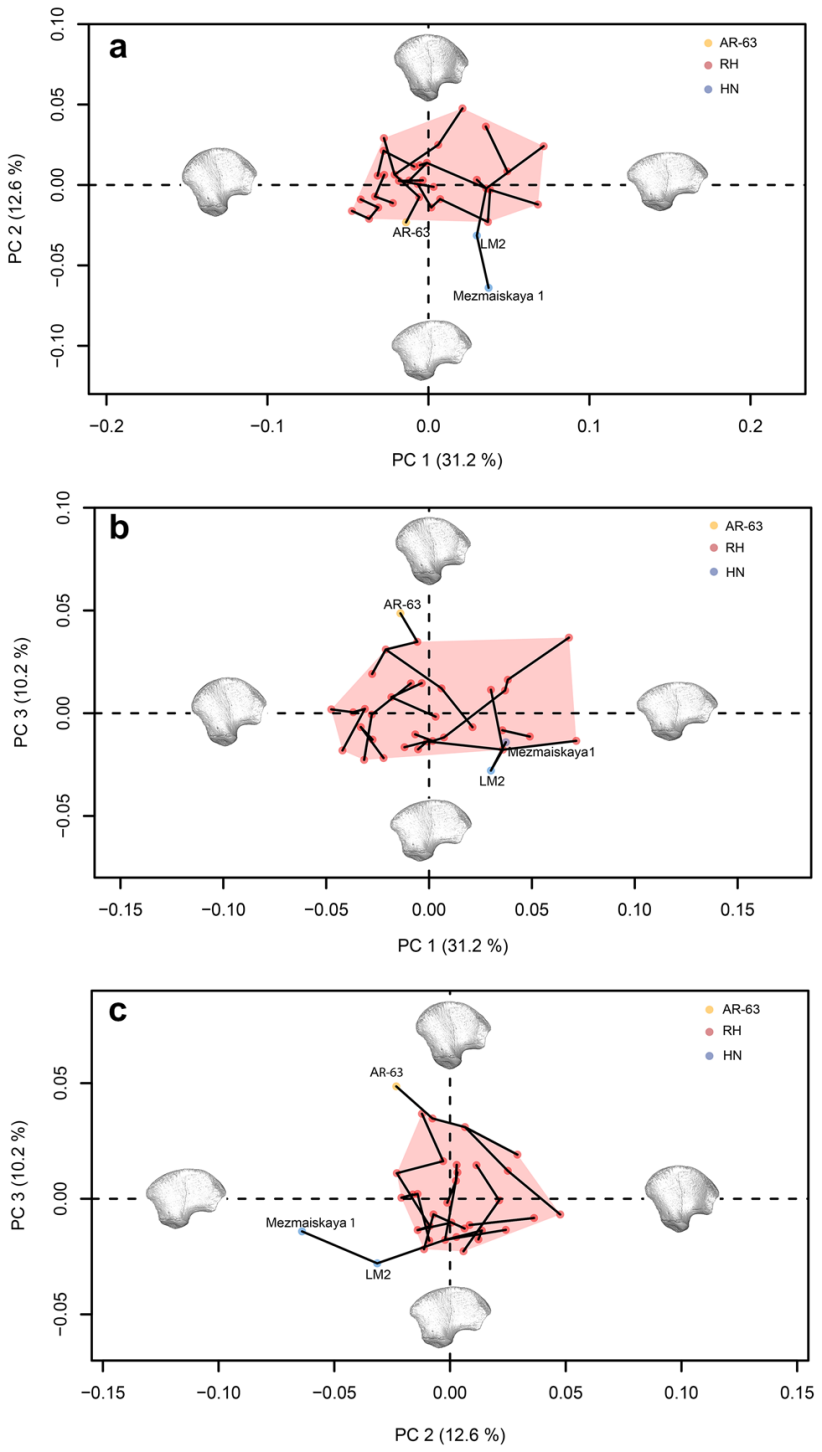
Principal component analysis in shape space of the general outline of the perinatal ilia showing the results of the Neighbor joining computed using Procrustes distances: (**a**) PC1 and PC2, (**b**) PC1 and PC3 and (**c**) PC2 and PC3. The red dots represent the recent perinatal sample (RH), the blue dots represent the perinatal Neanderthal sample (HN) and the yellow dot represents AR-63. A red convex hull is drawn for the RH. The black lines between individuals make the link between the nearest neighbors based on the inter-individual closest Procrustes distances. Surface warps representing the variation along each component are showed in lateral view at the positive and negative ends of each axis (±5 SD from the mean).

After defining the age group of perinatal individuals by performing a principal component analysis (PCA) in form space (taking into account size effects; Supplementary Information, Supplementary Methods 2 and Supplementary Fig. S2) we analyzed the distribution of the single age group of perinatal individuals within a PCA in shape space to discuss the taxonomic diagnosis of AR-63 (Fig. 4). We limited the PCA to the first three PCs which together represent 54% of the total variance. The two first PCs summarize 43.8% of the total variance (PC1: 31.2%; PC2: 12.6%) (Fig. 4a).

Along PC1, the three fossil specimens fall in the middle of the variability of RH, with the two Neanderthal neonates, LM2 and Mezmaiskaya 1(MZ1), being very close to each other. According to PC2, LM2 and MZ1 plot outside the RH variation in the negative values. MZ1 is the most distant while LM2 is much closer to the RH cluster. AR-63 is found on the periphery of the RH variation in the negative values.

To further explore the position of fossils relative to RH we analyzed their distribution according to PC3 reflecting 10.2% of the total variance (Fig. 4b). Along PC3, we observe that AR-63 and LM2 plot outside the RH variation. MZ1 is included within their variability but at the periphery. Relatively to the RH variation, the two Neanderthals plot in negative values at the opposite side of AR-63 plotting in positive values on PC3.

According to the plot of PC2 and PC3, we note that the three fossils are all excluded from the variability of the RH with AR-63 which is the closest, then LM2 and MZ1. On PC3, AR-63 shows the highest positive value and, as such, is clearly opposite of the two Neanderthals placed at the minimum end of the negative values with LM2 corresponding to the lowest value.

The computation of the extreme morphologies of the two first PCs allows us to visualize the shape variation along each axis (Fig. 4). With the three fossil specimens placed within the RH variability, PC1 does not reflect any taxonomic signal but illustrates inter-individual morphological differences unrelated to the slight different age-at-death between full-term and newborn individuals, as size only explains 17% of the variation along this axis (adjusted R-squared = 0.17, p-value = 0.007), or their geographical origin (Supplementary Information, Supplementary Fig. S3). The minimal values characterize individuals whose ilia present a greater height relatively to the anterior-posterior length and a concave anterior edge. On the contrary, the individuals placed within the maximum values of PC1 have a more extended anterior-posterior length and a straighter anterior edge.

**Figure 5:**
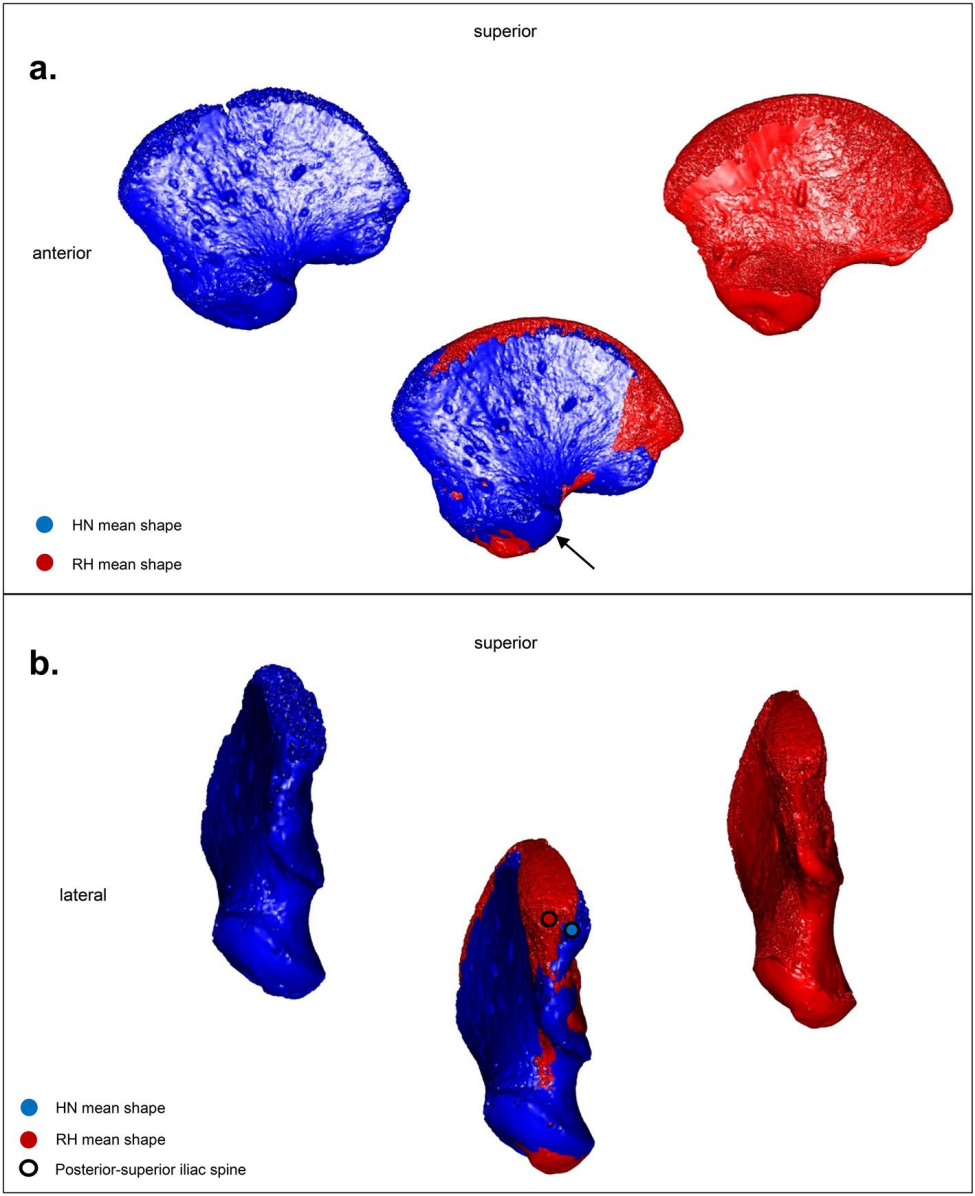
(**a**) Comparison between the mean shapes of the Neanderthals (HN, blue) and the recent perinatal individuals (RH, red) in lateral view. The superposition of the ilia highlights the differences between recent individuals and Neanderthals in the orientation of their acetabular area, more posterolateral (black arrow) among the latter. (**b**) Same comparison in posterior view. Note the distance between the posterior-superior iliac spines of the Neanderthal mean shape (blue dot) and the mean shape of recent individuals (red dot). The figure was generated using R software (version 4.1.2) http://www.R-project.org/ and Adobe Illustrator (version 4.1.2) https://www.adobe.com/products/illustrator.html.

PC2 discriminates individuals based on the outline of the acetabular area. In lateral view, the anterior half of this surface shows a more inferior orientation in the two Neanderthals accompanied by a more superior posterior half compared to the other specimens. By superimposing the Neanderthal mean shape calculated from the Procrustes coordinates of LM2 and MZ1, on that of the mean of the RH, it is clear, when positioning the ilium in lateral view, that the two Neanderthals have an acetabular area that is more posterolateral in orientation than that of the RH, for which it is placed anterolaterally (Fig. 5a). In addition, both Neanderthal ilia are characterized by a less prominent posterior-superior iliac spine compared to RH.

**Figure 6:**
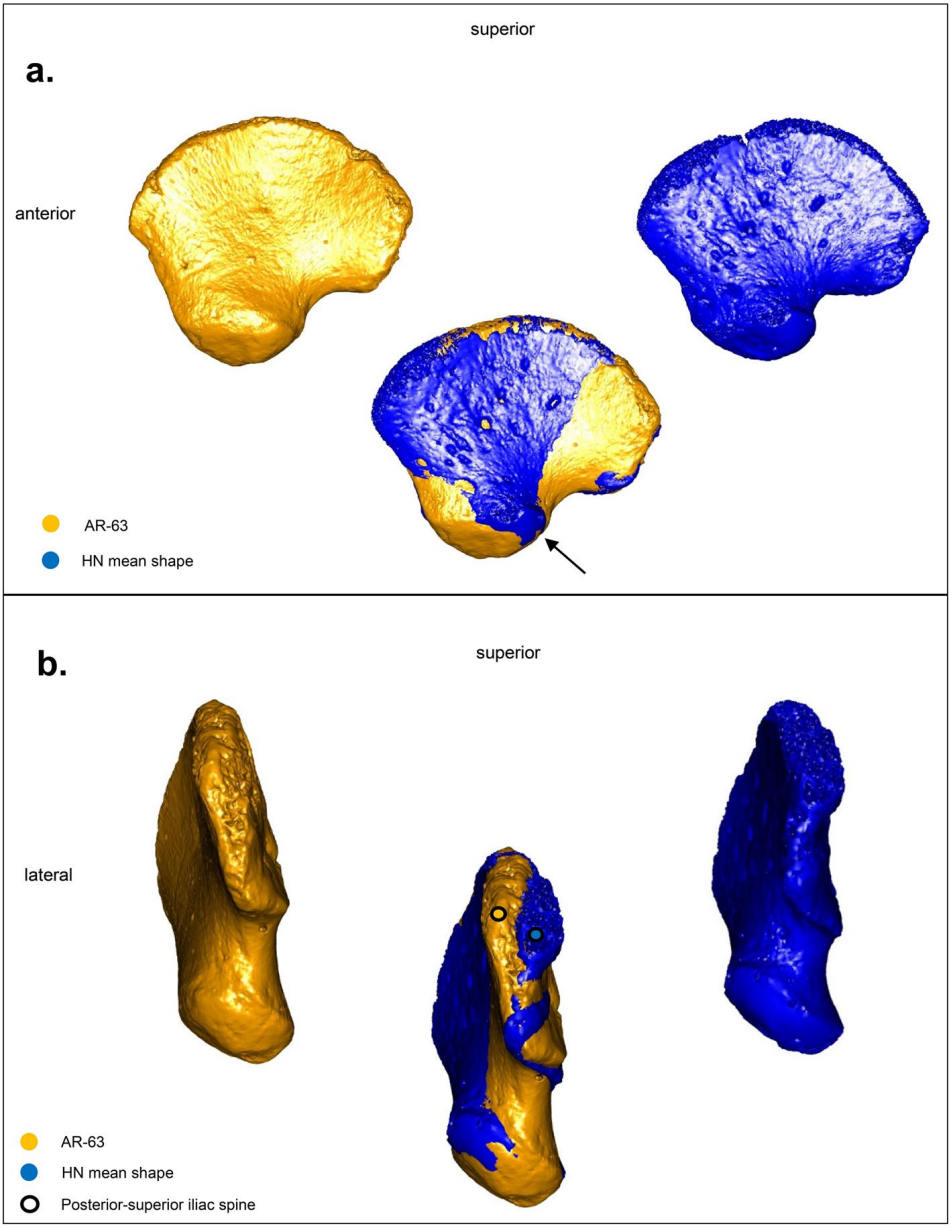
(**a**) Comparison between AR-63 shape (gold) and the Neanderthal mean shape (HN, blue), in lateral view. The superposition of the ilia highlights the shape differences between AR-63 and the Neanderthal individuals in the orientation of their acetabular area, more posterolateral (black arrow) among the latter. (**b**) Same comparison in posterior view. Note the distance between the posterior-superior iliac spines of AR-63 (gold dot) and the Neanderthal mean shape (blue dot) indicating a difference in their iliac crest curvature. The figure was generated using R software (version 4.1.2) http://www.R-project.org/ and Adobe Illustrator (version 4.1.2) https://www.adobe.com/products/illustrator.html.

**Figure 7:**
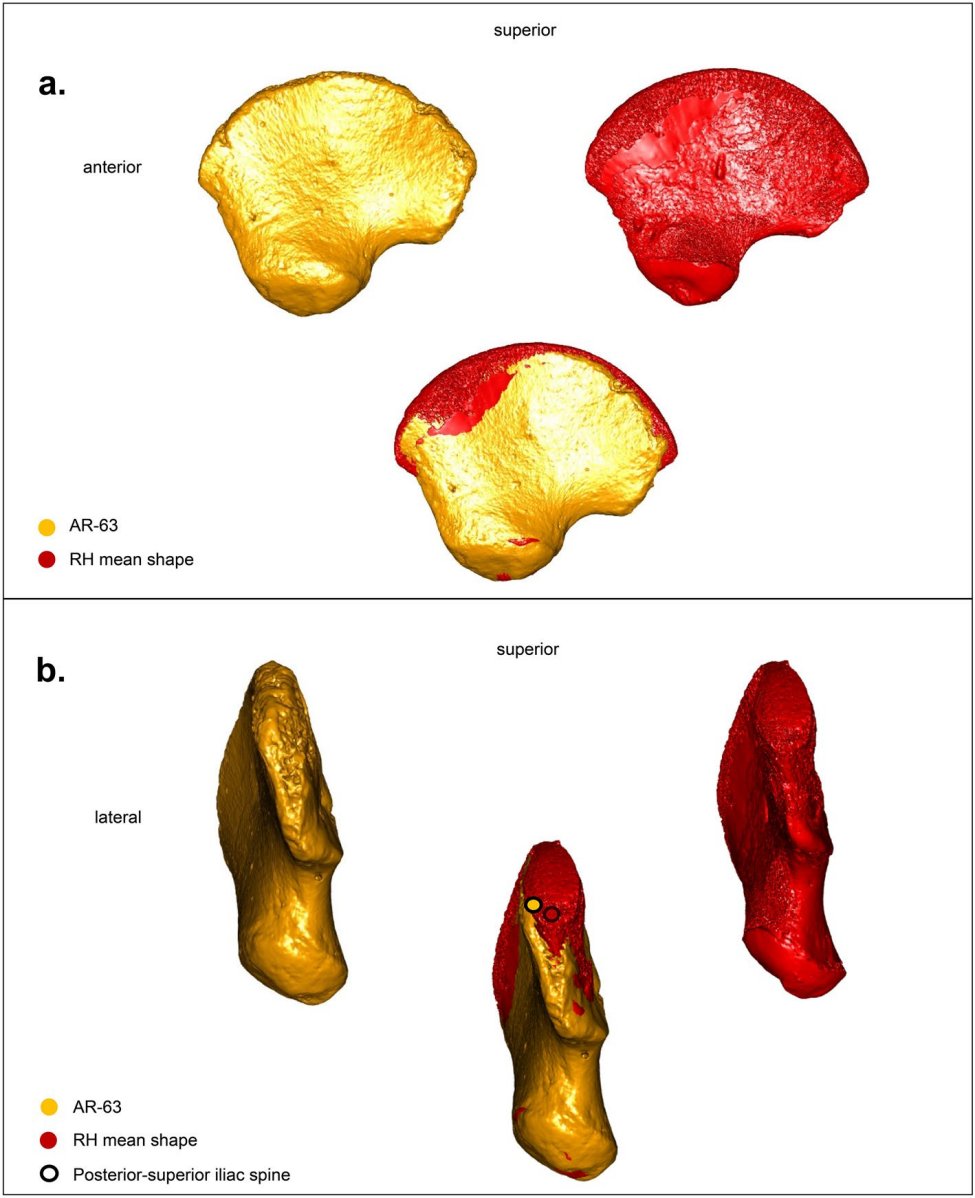
(**a**) Comparison between the AR-63 (gold) and the mean shape of the recent perinatal individuals mean shape (RH, red) in lateral view. The superposition of the ilia highlights the morphological proximity between AR-63 and the recent individuals, especially in the orientation of their acetabular area. (**b**) Same comparison in posterior view. Note the similar curvature of their iliac crest and the slight difference in the localization of their posterior-superior iliac spine. The figure was generated using R software (version 4.1.2) http://www.R-project.org/ and Adobe Illustrator (version 4.1.2) https://www.adobe.com/products/illustrator.html.

AR-63, placed at the periphery of the RH cluster, is clearly distinct from Neanderthals by a more anterolateral acetabular area (Fig. 6a), very similar to that of RH (Fig. 7a) but with a less prominent posterior-superior iliac spine than them.

PC3 reflects morphological differences linked to the iliac crest curvature. Superimposing the Procrustes coordinates of AR-63 on those of the Neanderthal mean shape allows us to detect a different curvature of the ilium (Fig. 6b). It is this curvature that influences the distribution of specimens along PC3 (Fig. 4b and c). In anterior, superior and posterior views this is visible by a difference in mediolateral orientation between the ilia. Indeed, when a portion of the iliac bone of AR-63 shows a medial inflection, the same region of the mean shape of the two Neanderthal neonates is oriented the opposite way and *vice versa*. This morphological distinction is stronger posteriorly with a significant distance observed between the two posterior-superior iliac spines, with that of AR-63 and RH oriented more laterally than that of the Neanderthals, which is characterized by a particularly curved posterior region (Figs. 5b and 6b). Moreover, it is interesting to note that AR-63 is distinct along PC3 from the RH sample. The superimposition of the Procrustes coordinates of AR-63 with those corresponding to the RH mean shape allows to detect a slight morphological distinction visible in the posterior part of the ilium with AR-63 exhibiting the most laterally oriented posterior-superior iliac spine of the whole sample (Fig. 7b).

In addition, the use of the Neighbor joining method allowed us to identify a closer morphological affinity of AR-63 to a RH than any other individual (Fig. 4). Finally, looking at the 3D distribution of our sample according to the first three PCs of our analysis (Supplementary Information, Supplementary Fig. S4) the three fossils stand out from the variability of RH and the morphology of AR-63 is opposite to that of the Neanderthals according to PC3 values. Relative to the RH variability, the Neanderthals are clearly distinct with MZ1 closer to LM2 than any other specimen.

## Discussion

The proximity observed between LM2 and MZ1 indicates a Neanderthal morphological homogeneity of the ilium in these two fossils representing groups separated chronologically by about 20,000 years and geographically by more than 3000 km (Fig. 1a). Moreover, by considering slightly older individuals (*circa* 2 years) such as the left ilium of La Ferrassie 8 (Southwest of France; Ref.^59^) or that of Dederiyeh 1^61^ we can observe that their shape is very close to the two perinatal Neanderthals. We observe that this morphological distinction on the ilium is mainly noticeable in the orientation of the acetabular area, which is more posterolateral in the two Neanderthals. In our opinion, this morphological feature is in line with what has been described on the ilium of the Neanderthal adult Kebara 2^68–70^. This morphological difference between Neanderthals and AMH has been proposed to be related to distinct posture and locomotor biomechanics in these two human groups^68,70^. In addition, Wolpoff^71^ describes, in relation to the pelvis of a much older fossil, that of *Australopithecus africanus* STS 14^72,73^ an orientation of the acetabulum more lateral to that of AMH. The "more laterally facing" acetabular area orientation has also been reported in chimpanzees (*Pan troglodytes*) relative to RH^74^. The identification of such a trait in ancient hominins, such as the Neanderthals, and great apes raises the question of the derived or plesiomorphic condition of the orientation of the acetabulum in RH. In any case, we consider that the more lateral orientation of the acetabulum in Neanderthal neonates testifies to its presence before any biomechanical or postural influences.

Furthermore, according to PC3, Neanderthal neonates are distinguished by a different curvature of the iliac bone, particularly in the posterior portion, more medially oriented in LM2 relative to AR-63 and RH. To our knowledge, this difference has never been described so precisely in immature Neanderthals although it was reported when comparing LM2 and a recent individual^29^.

If LM2 clearly appears outside the RH cluster according to PC3, this is not the case for MZ1 which is placed, admittedly at the periphery, but within the RH variability. This overlap could therefore indicate a variability of iliac curvature shared between Neanderthals and *Homo sapiens*. This feature will require further research, at least by attempting to increase the sample of RH.

Note, however, that the posterior portion of MZ1 is less well preserved than that of LM2, the posterior-superior iliac spine being absent, the estimation of the missing portions was computed based on the morphology of the whole sample, including fossil specimens, which may have influenced the position of the landmark placed on the posterior-superior iliac spine of MZ1 (see “Methods” and Supplementary Fig. S1). Indeed, by superimposing the Procrustes coordinates of MZ1 on those corresponding to the mean shape of RH, we observe, in posterior view, a shorter distance between their posterior-superior iliac spines (Supplementary Information, Supplementary Fig. S5a). This distance is greater when a RH ilium is compared to LM2 (Supplementary Information, Supplementary Fig. S5b), whose posterior-superior iliac spine, better preserved than that of MZ1, and not considered as a missing portion in our study, is more "medially offset". This is confirmed by superimposing the Procrustes coordinates of the two perinatal Neanderthals (Supplementary Information, Supplementary Fig. S5c).

Thus, we consider that based on the distribution of RH according to PC2 and PC3, the position of the two perinatal Neanderthals reflects the taxonomic distinction between these two groups. These results are in agreement with those indicating the presence of postcranial morphological differences between the two human groups from birth^59,62–64,66,75,76^.

Our analysis identifies morphological differences that distinguish AR-63 and the RH sample from the Neanderthal morphology. In AR-63 and the RH, the acetabular area is less posterolaterally oriented (Figs. 5a and 6a) and their posterior superior iliac spine is clearly more laterally oriented (Figs. 5b and 6b) than in the two Neanderthals. Therefore, we propose that the shape of the AR-63 ilium represents an AMH morphology.

Moreover, we were also able to identify a slight difference between AR-63 and the RH sample visible in the posterior part of the ilium, with AR-63 having a more laterally oriented posterior-superior iliac spine (Fig. 7b). Considering this slight difference highlighted by our study, we propose that AR-63 represents an individual from an early AMH lineage (likely from MIS 3 given what our knowledge on the human occupation of the GDR) for which the variation differed from RH. Similar results, on the position of a fossil relative to the variability of Neanderthals, Late Paleolithic and Holocene individuals, were recently described for a deciduous tooth uncovered in Grotte Mandrin (Rhone Valley, Southeast France)^77^. Morphological differences between UP and Holocene human groups have already been highlighted, for example in dental dimensions^78–80^. Similarly, genetic data from AMH fossils from Ust'-Ishim (Russia), Peștera cu Oase (Romania), Bacho Kiro (Bulgaria), and Zlatý kůň (Czech Republic) unearthed in Western Eurasia and predating 37 kya cal BP (Fig. 1), also shows a lack of genetic continuity between these ancient human groups and later Europeans^81–84^.

Regarding the chronological antiquity of AR-63, it would be interesting to include other fossil perinatal ilia from the UP. Based on previously published figures, the Epigravettian ilia of the Ostuni 1b fetus (Italy; Ref.^85^) presents in superior view a curvature of the iliac crest clearly closer to that of AR-63 than to that of LM2 and MZ1, a feature that we have described as discriminating between RH and Neanderthals. For the Gravettian, only the two unpublished newborns from Krems-Wachtberg (Austria; Ref.^86^) would be available, as to date, no human fossil ilium deriving from the different phases of the Aurignacian or Protoaurignacian has been discovered. Concerning older fossils, the left ilium of the perinatal Qafzeh 13 (Israel; ^87^), only partially well preserved, has an acetabular area with an anterolateral orientation on the basis of Figure 11 (Ref.^87^, p. 192), therefore closer to the morphology of our RH sample. Altogether, these previous descriptions suggest that the lack of knowledge about the variability of Neanderthal and ancient AMH perinatal ilia does not limit us in our taxonomic interpretation of the ilium of AR-63.

To date, research conducted on the transition between the MP and the UP in southwestern Europe considers that the Châtelperronian is a techno-complex of the beginning of the UP. According to some, it shows a Mousterian component only when overlying Mousterian layers, suggesting that these component primarily results from post-depositional admixture^10,19,20,38–42^. Moreover, this techno-complex shares many techno-typological traits with the Protoaurignacian industries due to the predominance of laminar production^88–96^. For others, however, the Châtelperronian is rooted in the Mousterian of Acheulean tradition^12,15,97^. This view is based on some other techno-typological similarities between the two techno-complexes, such as the presence of backed and elongated pieces or the use of direct percussion with a soft hammer and a similar geographical distribution of the two assemblages^98^. In this view, the Châtelperronian would therefore result from the evolution of local Neanderthal production either independently from any external influence or under the cultural influence of contemporaneous AMH. The first option implies an independent development of symbolic artifacts such as Châtelperronian body ornaments within Neanderthal populations^21,32,99,100^. The second implies direct or indirect contacts of late Neandertals with AMH bearing the early Aurignacian or other early UP assemblages resulting in acculturation and possible population admixture^11,13,27,101–104^.

If we assume that Neanderthals (acculturated or not) are the only makers of the Châtelperronian^14–18,21,24^ then the AR-63 ilium could be considered intrusive within the Xb2 level (hypothesis H1). This hypothesis would be consistent with the suggestion by some that layer admixture occurred at the GDR^10,19,20,38–42^. The bone could have come from overlying layer of the Aurignacian, as a result of human or large carnivore activities^42^.

Only a direct dating of the piece could resolve this issue^49^. However, taking into account the post-excavation history (including various manipulations, consolidations and probably casting^29^) and the very thin compact bone of the specimen, to date, no direct dating was undertaken.

If one assumes that AMH are the makers of the Châtelperronian^8–10,19,20,23,88–96,105^, then the AR-63 ilium could be *in situ* but all the Neanderthal remains of the layer should be intrusive and derived from the underlying Mousterian (hypothesis H2). We consider H2 unlikely. Although a large number (n=38) of Neanderthal human remains were unearthed at the GDR in association with Châtelperronian material, much less were discovered in the underlying Mousterian layer (n=6). This situation contrasts with that observed in the nearby Grotte du Bison where many Neanderthal remains were found in the late Mousterian levels (n = 50) and none in the Châtelperronian. Furthermore, the surface distribution of Neanderthal remains in the Châtelperronian layers at the GDR seems hardly compatible with H2^29^. Finally, and even more critically, H2 is at odds with the direct dating of one genetically identified Neanderthal from the Châtelperronian layer of the GDR matching the radiocarbon dates obtained in this layer^14^ and postdating the Mousterian layers at the site.

A third hypothesis (H3) could be that all human remains, both AR-63 and the Neanderthal fossils, are intrusive. Vertical movements, from top to bottom, in the case of AR-63, and from bottom to top, in the case of the Neanderthal pieces, would explain the presence of these remains within the Châtelperronian sequence. If such disturbances had occurred, they would have necessarily mobilized all the archaeological material, including the most characteristic Châtelperronian lithic pieces. However, none of the previous published studies that question the integrity of the archaeological levels of the GDR^10,19,20,38–42^ have documented such significant disturbances.

If we assume that the integrity of the Châtelperronian levels is as good as that of the underlying Mousterian and those of the overlying UP, and that the perinatal ilium is indeed contemporary with the Neanderthal human remains of layer Xb2, then AR-63 would attest the presence of AMH in this area of western Europe during the Châtelperronian period. The makers of the Châtelperronian could then be human groups where Neanderthals and AMH coexisted (hypothesis H4) or the GDR could have been occupied alternately by distinct human groups, makers of the same techno-complex (hypothesis H5).

If validated, H4 would bring strong support to the notion that the development of an UP like assemblage such as the Châtelperronian associated to Neanderthal makers at the time of the transition resulted from cultural diffusion or acculturation processes with possible population admixture between the two groups^13,104^.

## Conclusion

The 3D morphometric analysis of the perinatal ilium from the Châtelperronian layer Xb2 of the GDR compared to two perinatal Neanderthal ilia and the RH collections allows us to attribute AR- 63 to the AMH as well as to discuss the presence of features on the ilium that distinguishing AMH and Neanderthals from the perinatal period. In addition, the curvature of the iliac crest distinguishes the two perinatal Neanderthals from the other ilia analyzed in this study, a feature that has not been described in immature individuals in detail previously. It would be interesting to integrate the older individual La Ferrassie 8 in order to reconstruct the growth trajectories of the Neanderthal ilium and to apply surface semi-landmarks on the whole bone, such as what was recently done for the ilium of the immature individual *Homo naledi* (U.W. 102a-138; Ref.^106^). Clearly different from Neanderthal morphology, AR-63 also shows morphological peculiarities, such as its very "laterally offset" posterior-superior iliac spine, that exclude it from RH variability. This reflects, in our view, an ancient biologically modern phenotypic expression not previously documented within RH variability.

We are aware that these results will need to be complemented by an expanded study of newborn ilia from other RH populations and fossils from the late Upper Pleistocene and Early Holocene.AR-63 has been unearthed in the level Xb2, i.e., at the base of the Châtelperronian sequence of the GDR, within which 11 Neanderthal fossils were also found. We consider the most parsimonious hypothesis, in the absence of any direct absolute dating, that this ilium originates from the chronological period corresponding to the transition between the MP and UP in this region of western Europe, between 45 and 41 kya cal BP^14,49,52^. Only a direct dating of AR-63 would give reliable information on its antiquity. Until now, its taxonomic identification documents in an unprecedented way the population dynamics of AMH groups that settled the middle latitudes of Eurasia since at least 45 kya cal BP^107^. Furthermore, additional analyses must be conducted to discuss the archaeological integrity of the Châtelperronian sequence of the GDR such as what has been done at Saint-Césaire^23^. Indeed, it incites to undertake taphonomic and spatial studies of the GDR remains since it is now the only site delivering human remains in Châtelperronian layers for which these kind of studies have not been carried out. Finally, if we accept the presence, on the same territory and associated with the same techno-complex, Neanderthals and AMH, it becomes crucial to test the hypothesis of a potential AMH genetic contribution within the genome of the Neanderthal individuals from Arcy-sur-Cure (hypotheses H4 and H5). Although the existence of genetic introgressions from ancient population of African origin into Middle Pleistocene Neanderthal genomes have already been identified^108–110^ (but see also Ref.^111^), a similar phenomenon remains to document for the time period of the Neanderthal replacement in Europe.

## Methods

We selected the ilia (left or right, depending on the state of preservation) of 4 highly premature individuals, 32 full-term and newborn individuals and 9 young children (Table 1).

**Table 1:** Number of recent humans for each osteological collection according to the different sub-age groups included in this study.

Collection	Period	Highly premature individuals	Full-term individuals (perinatals)	Newborn (perinatasl)	Young children	Total
Necropolis 8B-51 of Sai island (Sudan)	1750-1500 BC	1	9	10	5	25
Church and cemetery of Saint-Ayoul de Provins (France)	XIII-XVIII century	3	7	6	4	20
Total		4	16	16	9	45

In our analysis we consider the full-term and newborn individuals as a single sub-age group that we call perinatals (see Supplementary Information, Supplementary Fig. S2).

The fossil sample consists of 2 Neanderthal individuals: LM2 and MZ1 and the individual AR-63. The very limited fossil comparative sample is due to the scarcity of perinatal remains from between the end of MIS 5 to MIS 4 and 3, and especially of well-preserved ones^62^.

### Fossil individuals: morphological description

#### Arcy 63 R Xb2 C12 3012 (AR-63)

AR-63 is a very well preserved right ilium of a perinatal individual, with a maximum height of 29 mm and a maximum length of 32 mm.

Regarding its contour morphology, in lateral or medial view (Fig. 3a), its anterior border, located below the anterior superior iliac spine, is straight. Superiorly it continues forming a convex arc between the two superior iliac spines corresponding to the iliac crest. The metaphyseal surface of the iliac crest is very slightly eroded in the posterior portion due to a small notch interrupting its convexity. In superior view, the curvature of the iliac crest is clearly marked, especially in its posterior portion which is laterally oriented (Supplementary Information, Supplementary Fig. S1). Observed in lateral or medial view, the posterior edge of the ilium, located between the posterior superior and the posterior inferior iliac spines, is straight. In posterior view, the inferior iliac spine is medially of the superior spine (Supplementary Information, Supplementary Fig. S1). In lateral or medial view, the greater sciatic notch is open and forms an obtuse angle. Regarding the inferior portion of the ilium, corresponding to the acetabular area, the inferior margin of the latter has a convex semicircular morphology, non-protruding. In lateral view, the superior delineation of the acetabular area is slightly convex and more open than that of its inferior margin so that, viewed laterally, its morphology resembles a "half-moon".

#### Le Moustier 2

LM2 is an almost complete skeleton of a Neanderthal newborn from the Lower shelter of Le Moustier, located in the Dordogne, about 45 km southeast of Périgueux (France) (Fig. 1a). Only the left ilium can be studied due to preservation. The LM2 skeleton was discovered in a pit dug from layer J to the top of the underlying layer H. Associated with a lithic techno-complex related to the typical Mousterian according to Peyrony (1930)^112^, a Levallois techno-complex according to Jaubert et al. (2011)^88^, Layer J has been dated by thermoluminescence and provided an age of approximately 40.3 kya +/− 2600 BP^113^. Electron spin resonance (ESR) dating of the underlying layer H yielded more recent mean ages^114^. In 2014, Higham et al. dated several bone remains from the Le Moustier sequence. Regarding the 5 pieces from layer J that provided a result, the dating fell between 45,100 +/− 2300 and 37.6 kya +/− 900 cal BP (Ref.^115^). The authors concluded that across the 20 horizons analyzed, the late Mousterian chronological interval was between 41,030 and 39,260 cal BP (at 95.4% probability). These latter results should be viewed with caution given the new understanding of the stratigraphy of this shelter^116^.

With a maximum height of 31 mm and a maximum length of 35 mm, the left ilium of LM2 is somewhat larger than AR-63. In lateral or medial view (Fig. 3b), LM2 has a concave anterior edge at the level of the center of the ilium below the anterior-superior iliac spine. This spine forms a less acute angle than that of AR-63. The slightly “blunt” appearance of the anterior-superior and posterior-inferior iliac spines of LM2 may be related to the slight erosion suffered by the entire metaphyseal surface of the iliac crest. Indeed, it is devoid of cortical bone and the cancellous bone is visible. Although slight, this taphonomic deterioration is more accentuated on a portion of the iliac crest, at the level of its first anterior half, forming a small notch that interrupts its natural convexity. Based on the morphology of the ilia of young Neanderthal children (*circa* 2 years old) with a well-preserved iliac crest, such as the left ilium of Dederiyeh 1 (Syria; Ref.^61^), we can state that this depression in the iliac crest of LM2 is related to a taphonomic alteration and not an anatomical feature. As viewed from superior, the curvature of its iliac crest clearly differs from that of AR-63 by being more straight (Supplementary Information, Supplementary Fig. S1). In lateral or medial view, the posterior margin of the ilium of LM2 has a relatively straight morphology although a very slight concavity is noticeable between the two posterior spines. In posterior view, the rim of LM2 is curved with iliac spines "oppositely positioned" relative to those of AR-63, namely, the lower spine "laterally offset" from the upper spine (Supplementary Information, Supplementary Fig. S1a). Seen laterally or medially, the angle formed by the greater sciatic notch of LM2, at about 90°, is much more closed than that of AR-63. The morphology of the inferior margin of the acetabular area, viewed laterally or medially, forms a convex arc relatively similar to that seen in AR-63. In contrast, in lateral view, the superior outline of the acetabular area of LM2 differs from that of AR-63 with an "elevation" of its posterior half so that the acetabular morphology is “teardrop”-shaped.

#### Mezmaiskaya 1

This specimen is part of a very well preserved skeleton of a Neanderthal neonate discovered in Mezmaiskaya Cave located in the northwestern Caucasus about 50 km south of Maikop (Russia) (Fig. 1a). The skeleton was found at the base of Level 3, dated to ca. 70-60 kya BP by ESR^60,117,118^. The left ilium bone is absent, so only the right was analyzed (Supplementary Information, Supplementary Fig. S1b).

With a maximum height of 32 mm and a maximum length of 38 mm, Mezmaiskaya 1 is more damaged than AR-63 and the left ilium of LM2, particularly at the anterior-superior margin, which is devoid of its iliac spine. The entire upper margin of the iliac crest is severely eroded, revealing cancellous bone down to the posterior-superior spine. Despite this alteration, it is possible, in superior view, to see the curvature of its iliac crest which is straight as in LM2 (Supplementary Information, Supplementary Fig. S1). The rest of the piece is better preserved and allows us to see several morphological similarities with the ilium of LM2. The posterior edge, viewed posteriorly, is similarly oriented with the lower spine "laterally offset" relative to the upper spine. In lateral view, the large sciatic notch forms an angle close to 90° and the acetabular area has a "teardrop" morphology (Supplementary Information, Supplementary Fig. S1b).

### Recent humans (RH)

The RH sample consists of a proto-historical and historical collections (Table 1) from recent excavations in which a significant number of individuals who died during the perinatal period have been discovered^67^. The proto-historical collection comes from the 8B-51 Classic Kerma necropolis from the Nile island of Saï (North Sudan, 1750-1500 B.C., programmed excavation, dir. F. Geus)^119,120^. We selected the ilia of 25 individuals (17 left and 8 right). The historical collection comes from a French medieval parish cemetery from Provins (France, 13th-18th centuries AD, excavation by INRAP and the University of Paris I, M. Guillon)^121^. The ilia of 20 individuals (17 left and 3 right) were included in this study.

Both collections are curated at the laboratory of PACEA laboratory (Bordeaux, France).

#### The 8B-51 necropolis of Saï Island

Located on the Nile between the second and third cataract in northern Sudan, this site has yielded a complex of 66 burials related to the end of the Classical Kerma period (1750-1500 B.C.), spread over an area of 150 m^2^ within an ancient alluvial terrace and buried under a layer of 20 to 40 cm of sediment^119,120^. Among these graves, 64 immature individuals were found, 54 of which had an estimated age-at-death between 24 and 46 weeks of amenorrhea based on the estimated stature from the maximum length of long bones^67^.

#### The church and the priory of Saint-Ayoul de Provins (France)

Located in the Parisian Basin, 77 km east of Paris, the church and parish cemetery of Saint-Ayoul de Provins were the subject of programmed excavations that uncovered in-place burials with a chronology ranging from the 13th to 18th centuries AD^122^. This site yielded a total of 111 primary individual burials including those of 90 children^123^. 35 individuals have an age at death that has been estimated to be between 26 and 46 weeks of amenorrhea^67^.

### Methods

As part of the constitution of our comparison sample, we based our age-at-death assessment methodology for very young individuals on the one chosen by one of us (C.P.)^67,124^, i.e. the method of Fazekas and Kósa (1978)^55^ revised by Sellier (cited in Ref.^125^). Since these formulae have been developed for various long bones, they can be applied to individuals from the fetal stage up to four years of age^126^ and provide confidence intervals. We then distinguish sub-age groups among the age group of individuals who died during the perinatal period *lato sensu*: highly premature individuals with mean age-at-death ranging between 24 and 34 completed weeks of amenorrhea, slightly premature and full-term individuals with mean age-at-death ranging between 35 and 40 completed weeks of amenorrhea and newborns with mean age-at-death ranging between 41 and 48 completed weeks of amenorrhea.

In an attempt to place the remains of AR-63 and the two Neanderthal neonate ilia within the morphological variability of RH ilia, we undertook a 3D geometric morphometric analysis that required the microtomographic acquisition of these specimens.

#### Microtomographic acquisitions

Arcy's ilium was scanned at the Department of Human Evolution of the Max Planck Institute Leipzig (MPI-EVA, Germany) using a tabletop micro-CT scanner (SkyScan 1173) with a resolution of 31 μm.

The acquisition of LM2 was performed at the Musée National de Préhistoire in Les Eyzies-de-Tayac (France) using an industrial μCT scanner (BIR ACTIS 225/300) from the Max Planck Institute for Evolutionary Anthropology (MPI-EVA, Leipzig Germany) with an isotropic voxel resolution of 70 μm^127^.

The 3D scanning of MZ1 was performed at Pokrovskaya Hospital in St. Petersburg (Russia) using helical CT (CT; beam collimation, 1 mm; pitch, 1; slice reconstruction increment, 0.3 to 0.5 mm)^118^.

Finally, the RH ilia were scanned at the PLACAMAT platform (UMS 3626, University of Bordeaux, France) using X GE™ V/TOME/X S microtomographic equipment with a resolution of 30 μm.

#### 3D data processing and geometric morphometrics analysis

**Figure 8:**
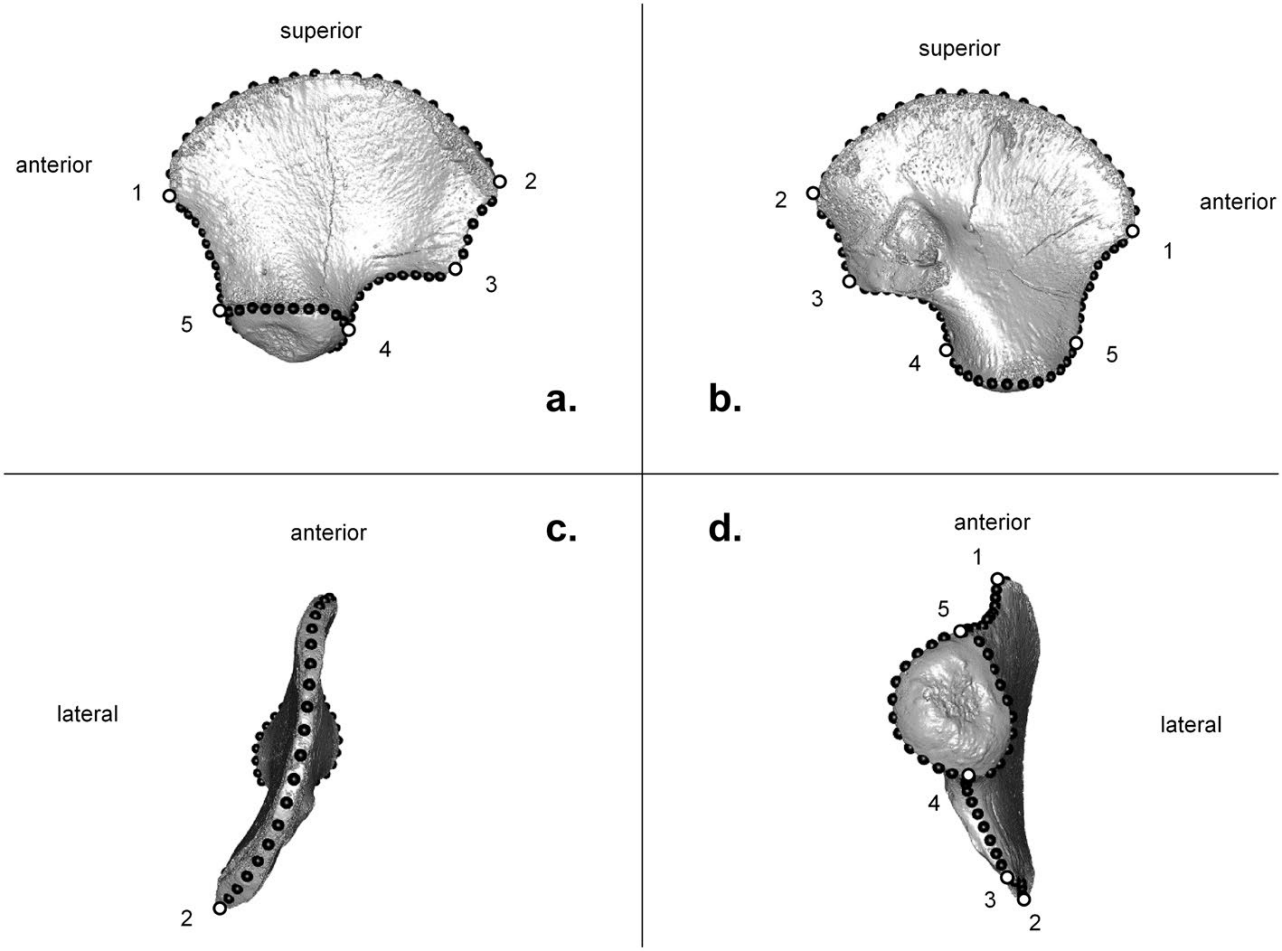
Landmarks template applied to a left ilium of a recent perinatal individual. (**a**) lateral view, (**b**) medial view, (**c**) superior view, (**d**) inferior view. White spheres numbered 1 to 5 correspond to fixed landmarks and black spheres are semilandmarks delimiting the curves described in Table 2.

After importing the image stacks into Avizo^®^ 7.0.1. software (Visualization Sciences Group Inc.), the ilium surfaces were first segmented using the density based thresholding using the histogram tool, then they isolated using the tool extract subvolume. From these surfaces, the contour of the iliac bone, when oriented in lateral or medial view, and that of the acetabular area were quantified by manually applying a template of 72 landmarks (5 fixed landmarks and 67 semilandmarks) on each ilium according to a protocol available in Supplementary Information, Supplementary Methods. Using the Avizo^®^ landmark module, anatomical landmarks were placed on the iliac spines: anterior-superior, posterior-superior and posterior-inferior, and two others were positioned anterior-posteriorly on either side of the acetabulum. Using the Avizo^®^ B-spline function, the semilandmarks were placed on the outline of the iliac bone through the outline of the acetabular area (Fig. 8 and Table 2).

**Table 2:** Landmark template description applied on ilia.

Number	Type	Description
1	Fixed landmark	Anterior superior iliac spine
2	Fixed landmark	Posterior superior iliac spine
3	Fixed landmark	Posterior inferior iliac spine
4	Fixed landmark	The maximal convexity posterior point of the acetabular outline
5	Fixed landmark	The middle anterior point at the base of the acetabular bony tongue
Set 1 (20 points between 1 and 2)	Curve semilandmarks	Iliac crest
Set 2 (5 points between 2 and 3)	Curve semilandmarks	Posterior iliac edge
Set 3 (10 points between 3 and 4)	Curve semilandmarks	Greater sciatic notch
Set 4 (12 points between 4 and 5)	Curve semilandmarks	Medial outline of the acetabulum
Set 5 (10 points between 4 and 5)	Curve semilandmarks	Lateral outline of the acetabulum
Set 6 (10 points between 5 and 1)	Curve semilandmarks	Anterior edge of the ilium

See the protocol available in Supplementary Information, Supplementary Methods for details.

The landmark coordinates were then imported and processed in RStudio^128^ using the Morpho^129^ and Geomorph^130^ packages. Missing landmarks were estimated, for some individuals with a slightly eroded iliac crest outline, using the thin-plate spline (TPS) interpolation function (Morpho package v. 2.8; Ref.^129^). After mirroring the landmark configurations of the 15 right ilia, due to the higher number of left ilia in our comparative sample, the bending energy was minimized by sliding the semilandmarks which can then be considered homologous^131–133^. The landmark coordinates were then converted to shape variables via a generalized Procrustes analysis (GPA; Refs.^134–136^). Results of the PCA were visualized by computing extreme shapes on each axis (±5 SD from the mean).

A PCA was conducted to visualize the distribution of specimens in form space and shape space to analyze their size and shape variation^137–139^ and discuss the taxonomic attribution of the right ilium of the Châtelperronian perinatal AR-63. Finally, to identify which individuals are most similar in shape to AR-63 we used the Neighbor joining method between pairwise Procrustes distances to identify nearest neighbors based on shape similarity^140^.

### Supplementary Information

Supplementary Information.

## Data Availability

The authors declare that all data supporting the conclusions of this study are present in the article and in the supplementary information file. The corresponding author is willing to share additional data upon reasonable request.
