# Supplementary material for: Anatomically modern human in the Châtelperronian hominin collection from the Grotte du Renne (Arcy-sur-Cure, Northeast France)
Source: Sci Rep. 2023 Aug 4;13:12682. doi: 10.1038/s41598-023-39767-2 (PMC10403518; doi:10.1038/s41598-023-39767-2)
Supplement: Supplementary file 1 — Supplementary Information. [file 41598_2023_39767_MOESM1_ESM.pdf]

Supplementary Information:

Anatomically modern human in the Châtelperronian hominin collection from the Grotte du Renne (Arcy-sur-Cure, Northeast France)

Arthur Gicqueau, Alexandra Schuh, Juliette Henrion, Bence Viola, Caroline Partiot, Mark Guillon, Liubov Golovanova, Vladimir Doronichev, Philipp Gunz, Jean-Jacques Hublin, and Bruno Maureille

Supplementary Methods

1-3D landmarking protocol to quantify the outline morphology of ilia of individuals deceased during the perinatal period

1.1. Orientation of the ilium in a 3D space.

- How we define the **medial and lateral view** (fig. 1) of the ilium: the bone must be orient with the iliac crest appearing superiorly, the acetabulum inferiorly, the anterior and posterior on the left or the right.
  - Anterior-posteriorly: as much as possible, the bone must be oriented so that it would be possible to visualize the maximal anterior posterior length of the ilium.
  - Superior-inferiorly: the ilium must be placed so that the line following the middle of the iliac crest thickness must appears as being the superior outline of the bone.

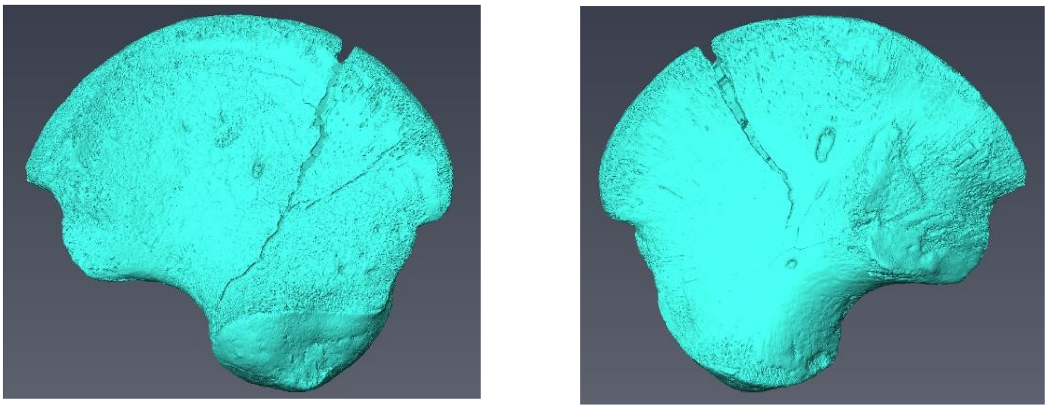

Fig. 1. Lateral (left) and medial (right) view.

The figures were generated using Avizo® software (version 7.0.1) www.vsg3d.com.

- How we define the **anterior view** (fig. 2):
  - Medio-laterally: the front edge between the anterior superior iliac spine and the anterior inferior iliac spine must appear vertically in the foreground in all its vertical lenght and the ilium’s posterior part should not appear on the left or the right side but must be masked by the acetabulum.

- Superior-inferiorly: the ilium must be placed so that the “acetabular bony tongue”, corresponding to the future anterior inferior iliac spine epiphysis (Scheuer and Black, 2004) must appears vertically in the foreground in all its vertical length. The posterior ilium’s part must not be visible under the acetabulum.

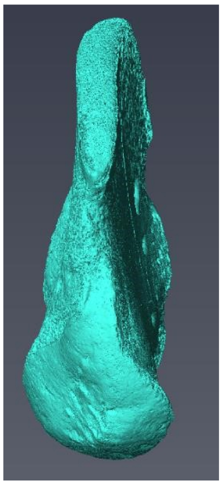

Fig. 2. Anterior view.

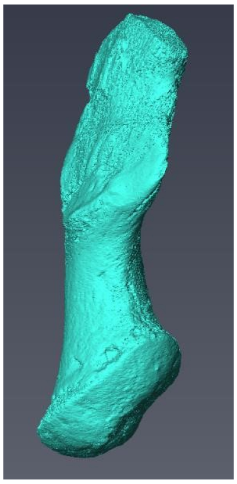

Fig. 3. Posterior view.

The figures were generated using Avizo® software (version 7.0.1) www.vsg3d.com.

- How we define the **posterior view** (fig. 3):
    - Medio-laterally: the greater sciatic notch must appear vertically in all its vertical length and the anterior ilium’s part bearing the anterior superior iliac spine should not appear on the left or the right side but must be masked by the acetabulum.
    - Superior-inferiorly: the ilium must be oriented in a way that the posterior inferior side of the iliac crest should not overlap the greater sciatic notch and the anterior ilium’s part bearing the anterior superior iliac spine should not be visible under the acetabulum.

1.2. Definition of landmarks.

- **Landmark 1: Anterior superior iliac spine.**

⇨ Place the ilium in **anterior view**, landmark at the middle of the crest thickness just above the tip corresponding to the most anterior inferior part of the iliac crest (fig. 4).

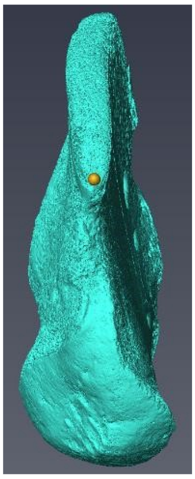

Fig. 4. Landmark 1.

- **Landmark 2: Posterior superior iliac spine.**

⇨ Place the ilium in **medial view**, from landmark 1 measure the maximal iliac length using the 3D measure tool (fig. 5). Place the point at the end of the segment traced (fig. 6).

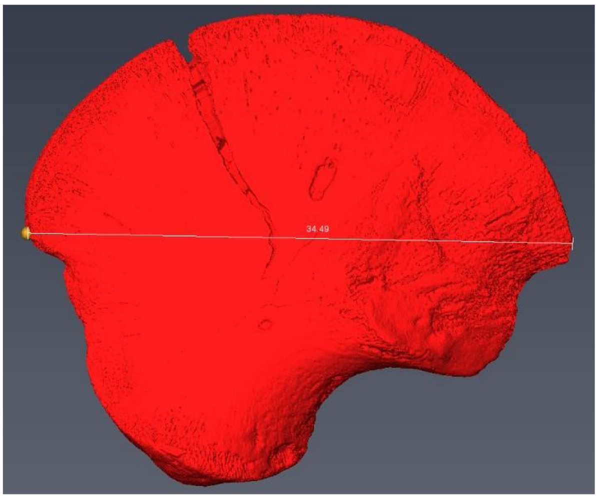

Fig. 5. Measure of the maximal iliac length.

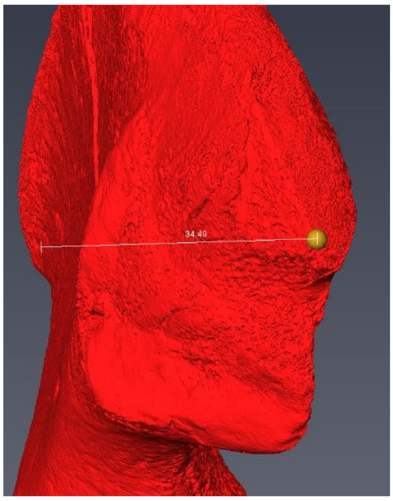

Fig. 6. Landmark 2

The figures were generated using Avizo® software (version 7.0.1) www.vsg3d.com.

- **Landmark 3: Posterior inferior iliac spine.**

⇨ Place the ilium in **medial view**. Firstly, compute the centroid point between landmark 1 and landmark 2 (fig. 7) and then compute the centroid point between the first centroid point computed and landmark 2 (fig. 7). From this second centroid point, measure the most important distance at the level of the posterior inferior region using the 3D measure tool. Place the point at the end of the segment traced (fig. 7).

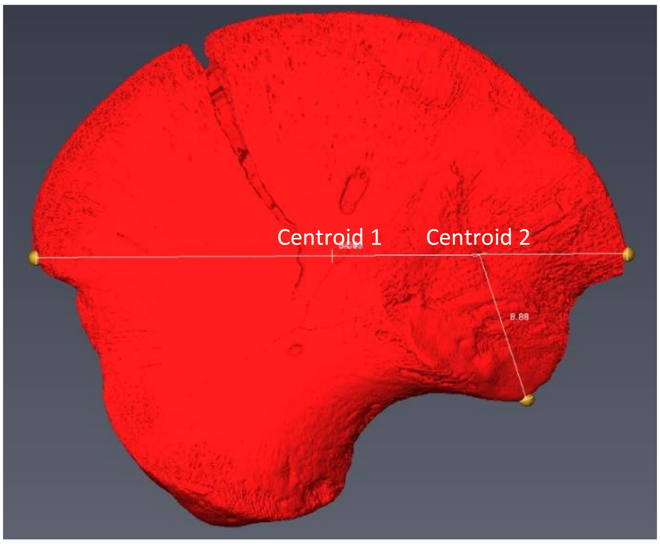

Fig. 7. Landmark 3.

- **Landmark 4**: **The maximal convexity posterior point of the acetabular outline.**

⇨ Place the ilium in **inferior view** in a way that the acetabulum must appears on his inferior face in the foreground with landmark 3 appearing superiorly and landmark 1 inferiorly (fig. 8). As much as possible, orient the acetabular area according to its maximal length with the lateral side tangent to the iliac crest. Place the landmark at the level of the maximal convexity formed by the acetabular outline, here visible superiorly, at the level of the greater sciatic notch (fig. 8).

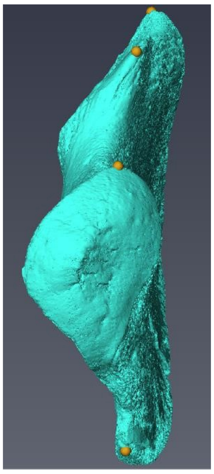

Fig. 8. Landmark 4.

The figures were generated using Avizo® software (version 7.0.1) www.vsg3d.com.

- **Landmark 5: The middle anterior point at the base of the acetabular bony tongue.**

⇨ Place the ilium in **lateral view**. Landmark at the meeting point between the acetabulum outline and the anterior edge (fig. 9). Then, orient the ilium in **anterior view,** the point appears on the right or the left (fig. 10). From landmark 1, draw a vertical straight line perpendicular to the bottom of your screen (fig. 11). Move the point placing it perpendicularly to the straight line relative to its first position (fig. 12).

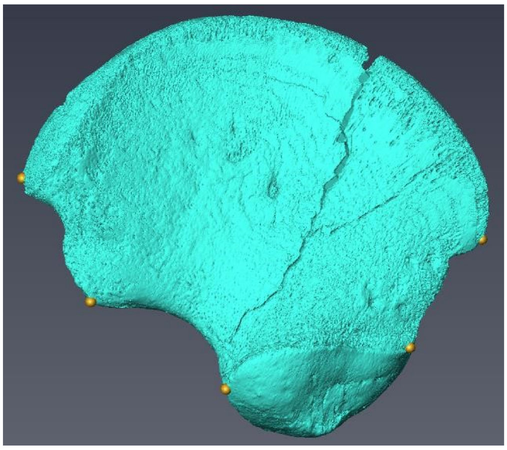

Fig. 9.

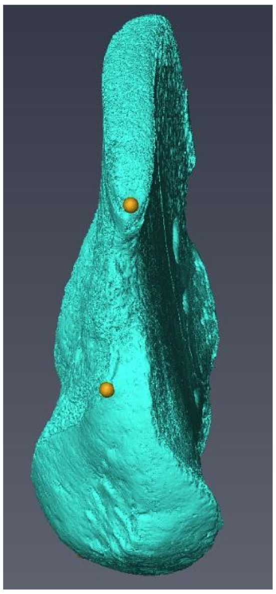

Fig. 10.

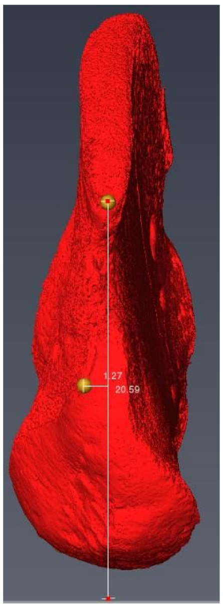

Fig. 11.

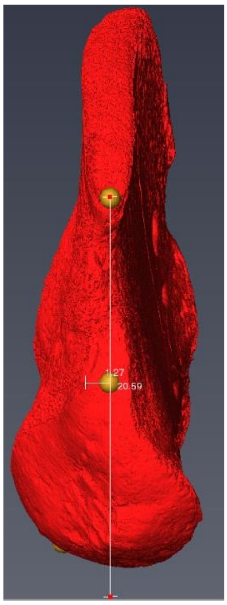

Fig. 12.

The figures were generated using Avizo® software (version 7.0.1) www.vsg3d.com.

1.3. Definition of semi-landmarks.

- **Set 1 : the iliac crest.**

⇨ From landmark 1 to landmark 2, place the set (20 semi-landmarks) following the middle of the superior iliac crest thickness (fig. 13).

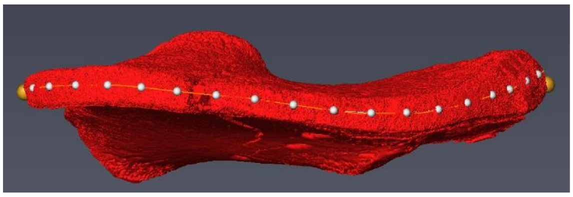

Fig. 13. Set 1 (superior view).

- **Set 2: the posterior iliac edge.**

⇨ From landmark 2 to landmark 3, place the set (5 semi-landmarks) following the posterior iliac edge (fig. 14).

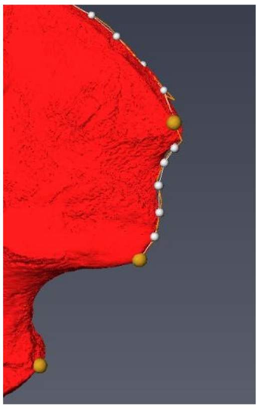

Fig. 14. Set 2 (medial view).

The figures were generated using Avizo® software (version 7.0.1) www.vsg3d.com.

- **Set 3: the greater sciatic notch.**

⇨ Firstly, orient the bone in **medial view**, from the centroid point between landmark 1 and landmark 2, computed previously, measure the shorter distance with the greater sciatic notch to detect the maximal concavity point (fig. 15). Place a point at the end of the segment traced (fig. 16). Then, in **posterior view**, draw a vertical straight line between landmark 3 and the maximal concavity point and place 6 semi-landmarks following the straight line (fig. 17). Do the same procedure placing 4 semi-landmarks between the maximal concavity point and landmark 4 (fig. 18).

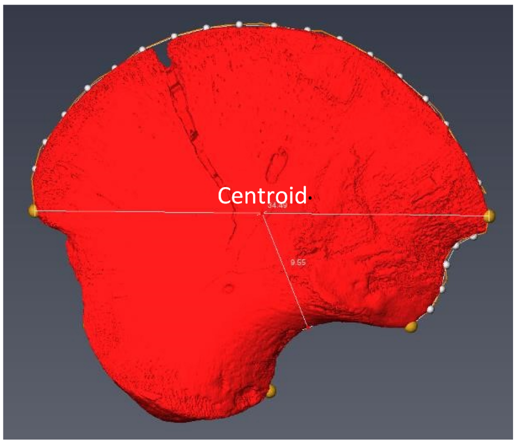

Fig. 15.

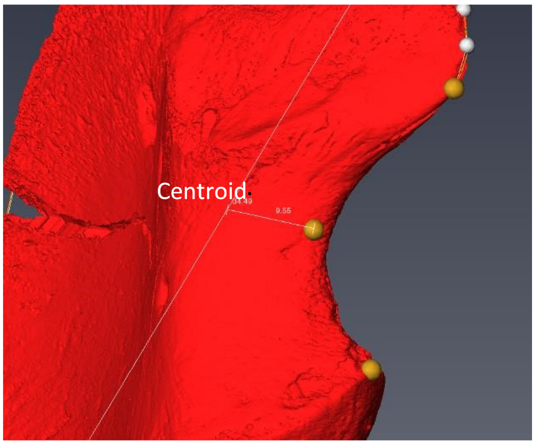

Fig. 16.

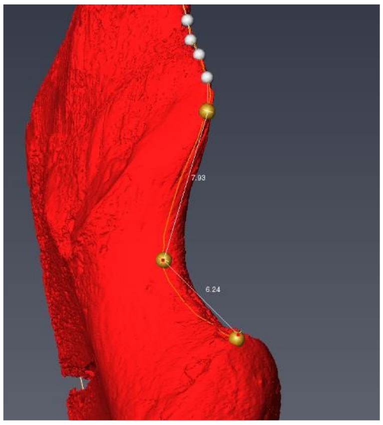

Fig. 17.

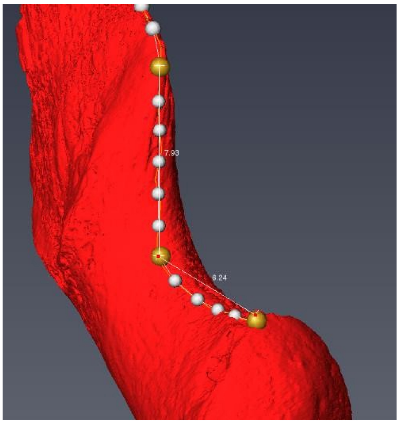

Fig. 18.

The figures were generated using Avizo® software (version 7.0.1) www.vsg3d.com.

- **Set 4: medial outline of the acetabulum.**

⇨ In **medial view**, from landmark 4 to landmark 5, place the set (12 semi-landmarks) following the acetabulum outline (fig. 19).

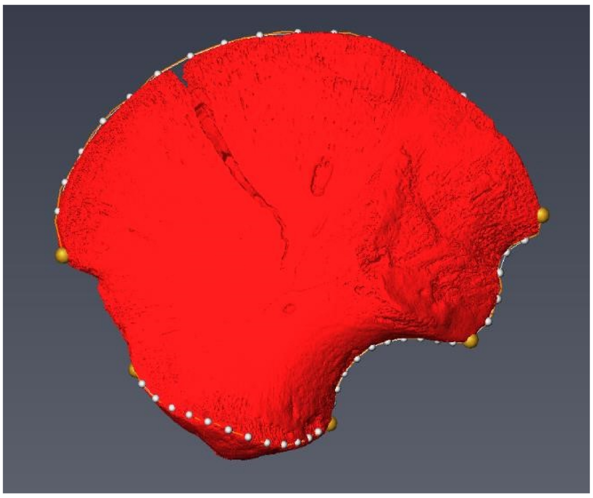

Fig. 19. Set 4.

- **Set 5: lateral outline of the acetabulum.**

⇨ In **lateral view**, from landmark 4 to landmark 5, place the set (10 semi-landmarks) following the acetabulum outline (fig. 20).

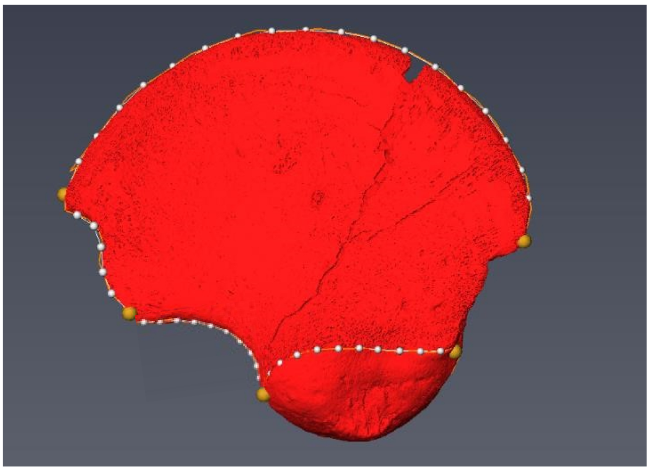

Fig. 20. Set 5.

The figures were generated using Avizo® software (version 7.0.1) www.vsg3d.com.

- **Set 6: anterior edge of the ilium.**

⇨ In **anterior view**, from landmark 5 to landmark 1, place the set (10 semi-landmarks) following firstly the middle of the acetabular bony tongue passing by the tip of the tongue and then, following the anterior edge until landmark 1 (fig. 21).

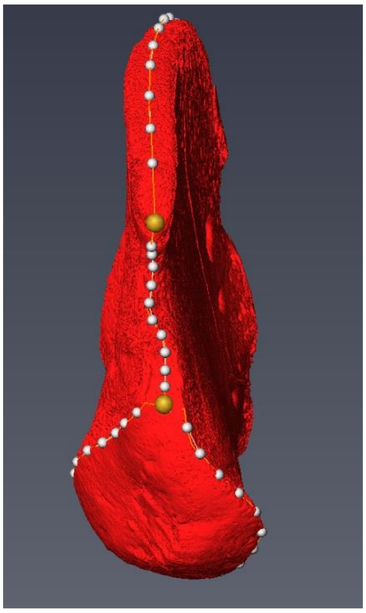

Fig. 21. Set 6.

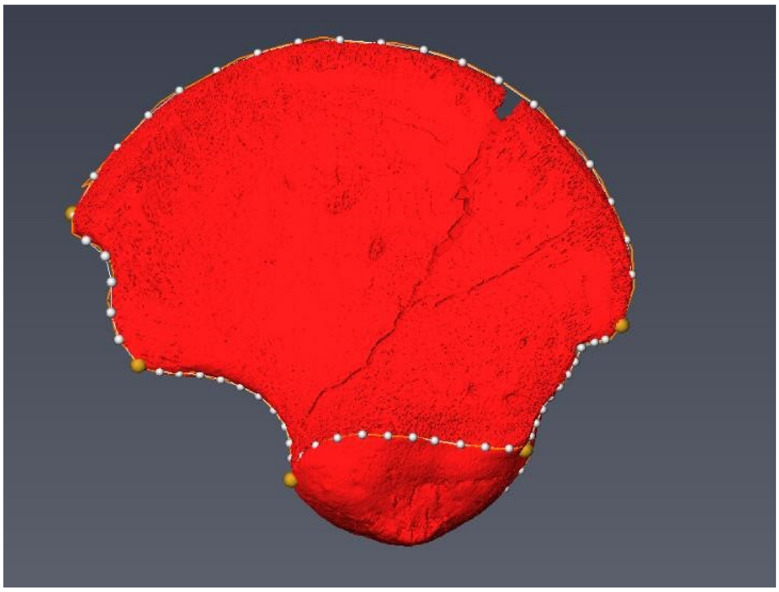

Fig. 22. Ilium fully landmarked (lateral view).

The figures were generated using Avizo® software (version 7.0.1) www.vsg3d.com.

2-Method used to define the age group of perinatal individuals

We first performed a principal component analysis (PCA) in form space, taking into account size effects, for our entire sample (fossil and recent including all four age groups; see "Methods" and Table 1), to observe the distribution of recent individuals (RH) by age-at-death and that of the three fossils relative to them (Supplementary Information, Supplementary Fig. S2). The two first principal components (PCs) account for 96.1% of the variability. The distribution by age groups clearly distinguishes highly premature individuals and young children from full-term and newborn individuals according to PC1 reflecting 94.4% of the variability. However, taking into account these sub-age groups (see "Methods") there is not such important distinction in the morphology of the ilium between full-term and newborn individuals. Due to the overlap observed along PC1 between several individuals of these two sub-age groups, reflecting a certain homogeneity in the shape of the ilium between 35 and 48 completed weeks of amenorrhea, we decided to consider them in this study as a single cluster that we decide to call perinatal individuals. According to PC2 which describes 1.7% of the variation, none of these sub-age groups are distinguishable. According to PC1, the three fossils clearly fit within the variability of RH.

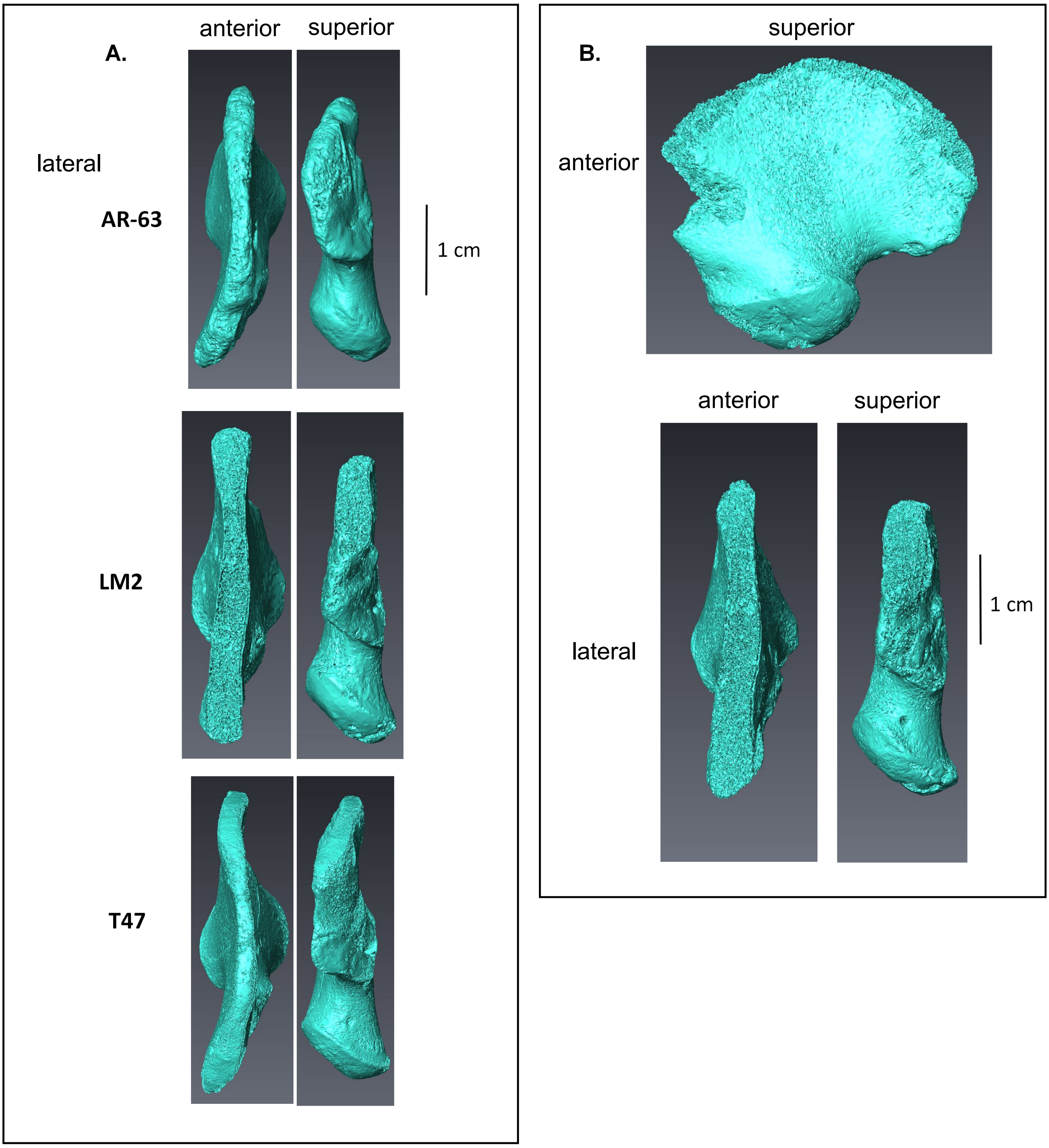

Supplementary Figure S1. A) Superior (left) and posterior (right) view of the perinatal right ilium (mirrored in left) of AR-63 (above), the left ilium of the perinatal Neanderthal Le Moustier 2 (LM2; middle) and the left ilium of the perinatal present-day individual T47 (bottom). B) Lateral (above), superior (bottom left) and posterior (bottom right) view of the perinatal Neanderthal left ilium of Mezmaiskaya 1 (MZ1) whose the anterior part is missing. Due to the small size of the perinatal Neanderthal ilia sample (HN), we decided to reconstruct the missing data of MZ1 from our total sample, which includes mostly recent perinatal individuals (RH) whose average morphology probably influenced the position of the missing landmarks on the anterior portion of MZ1. Despite this constraint, MZ1 is clearly distinguishable from RH according to PC2, and appears closer to LM2 than any other individual, indicating the existence of morphological differences sufficiently marked to distinguish HN from RH perinatal ilia (Fig.4a and 4c).

The figure was generated using Avizo® software (version 7.0.1) www.vsg3d.com and Adobe Illustrator (version 4.1.2) https://www.adobe.com/products/illustrator.html.

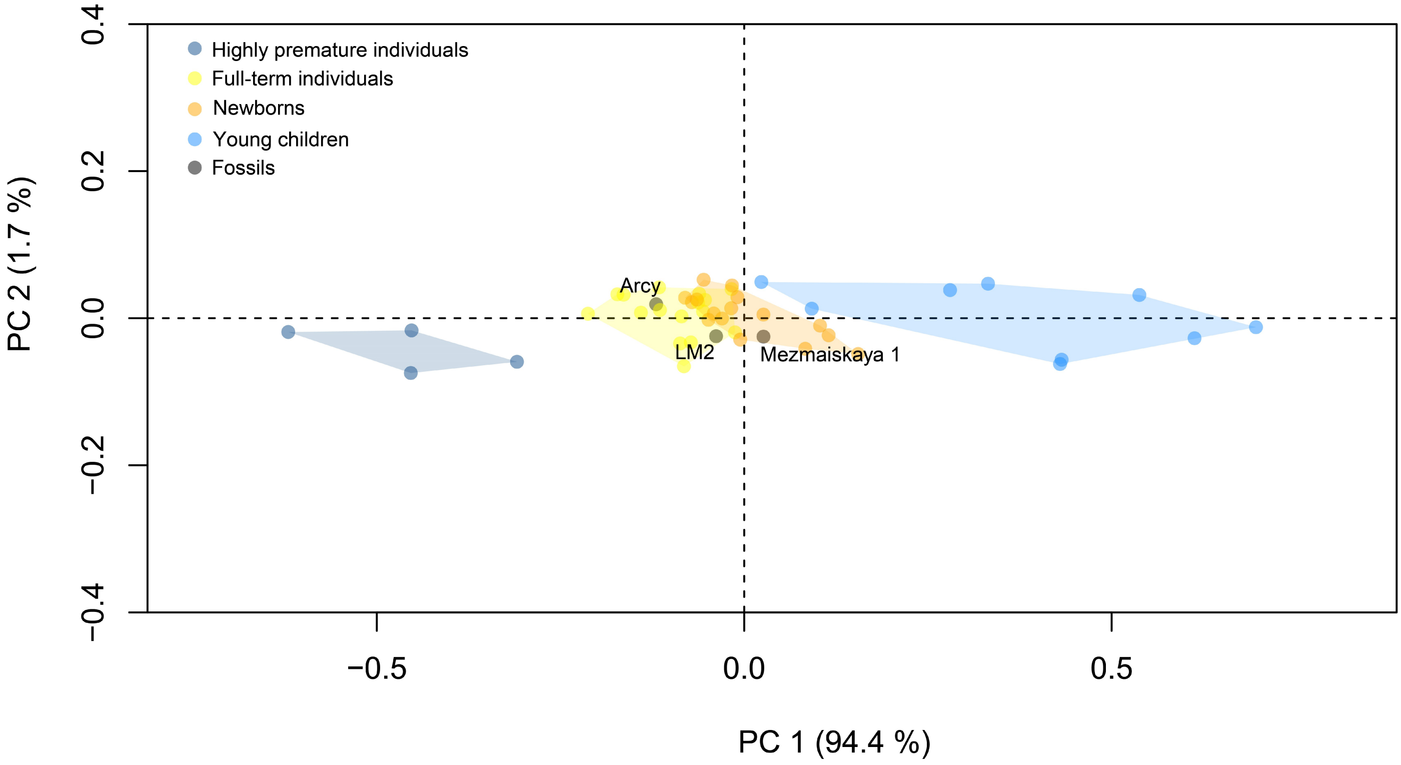

Supplementary Figure S2. Principal component analysis of the form space: PC1 and PC2 of the general outline morphology of the ilia of fossils (black dots) and proto-historical and historical individuals: highly premature individuals (dark blue), full-term individuals (yellow), newborns (orange) and young children (light blue). The distribution of the individuals in the form space according to PC1 highlight that the two sub-age groups, full-term individuals and newborns, present a certain homogeneity in the shape and size of the ilium allowing to consider them, in this study, as a single sub-age group that we call perinatal individuals.

The figure was generated using R software (version 4.1.2) http://www.R-project.org/ and Adobe Illustrator (version 4.1.2) https://www.adobe.com/products/illustrator.html.

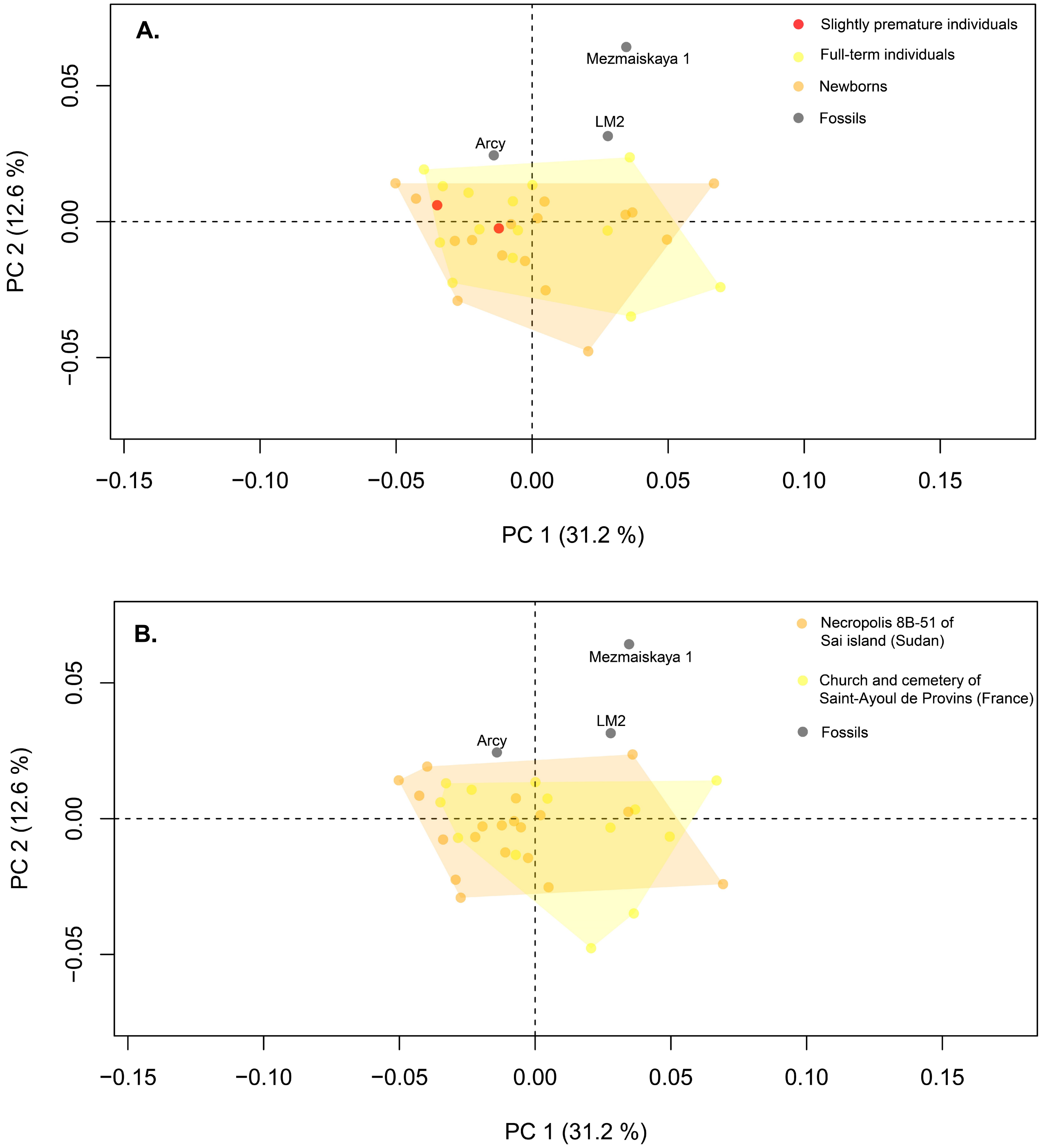

Supplementary Figure S3. Principal component analysis of the shape space: PC1 and PC2 of the general outline morphology of the ilia of fossils (black dots) and proto-historical and historical individuals according to A) their estimated age-at-death : slightly premature individuals (red dots), full-term individuals (yellow), newborns (orange) and B) their geographical origin : individuals from the Necropolis 8B-51 of Sai Island (Sudan) (orange) and individuals from the church and cemetery of Saint-Ayoul de Provins (France) (yellow). In both graphs and according to PC1, the overlap observed between the different groups indicate that the distribution of the individuals is not influenced by their slightly different age-at-death nor by their geographical origin.

The figure was generated using R software (version 4.1.2) http://www.R-project.org/ and Adobe Illustrator (version 4.1.2) https://www.adobe.com/products/illustrator.html.

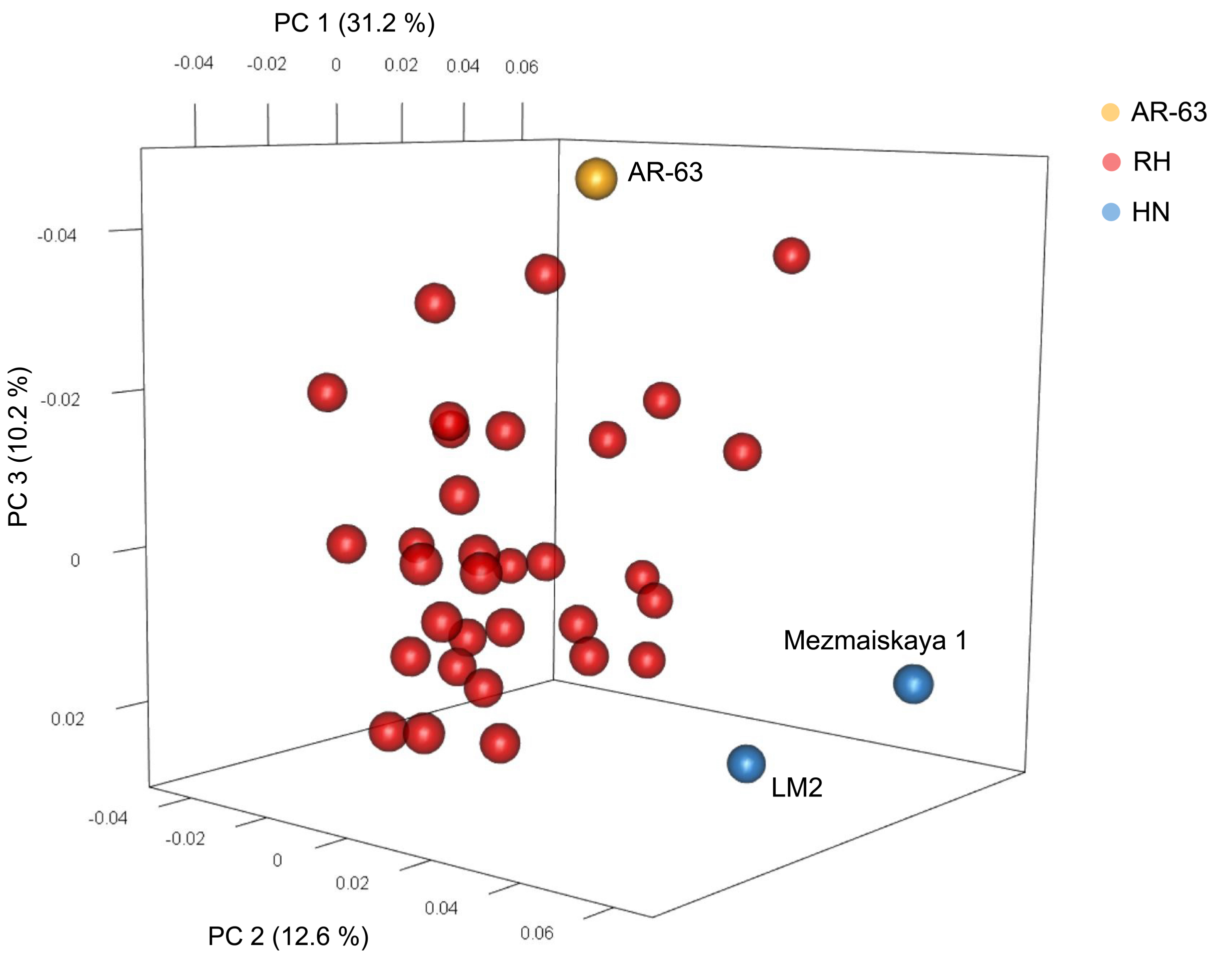

Supplementary Figure S4. Three-dimensional view of the principal component analysis (PC1, 2 and 3) of the general outline shape of neonate ilia. Gold dot: AR-63; blue dots: Neanderthals (HN) Le Moustier 2 (LM2) and Mezmaiskaya 1 (MZ1); red dots: recent individuals (RH).
The figure was generated using R software (version 4.1.2) http://www.R-project.org/ and Adobe Illustrator (version 4.1.2) https://www.adobe.com/products/illustrator.html.

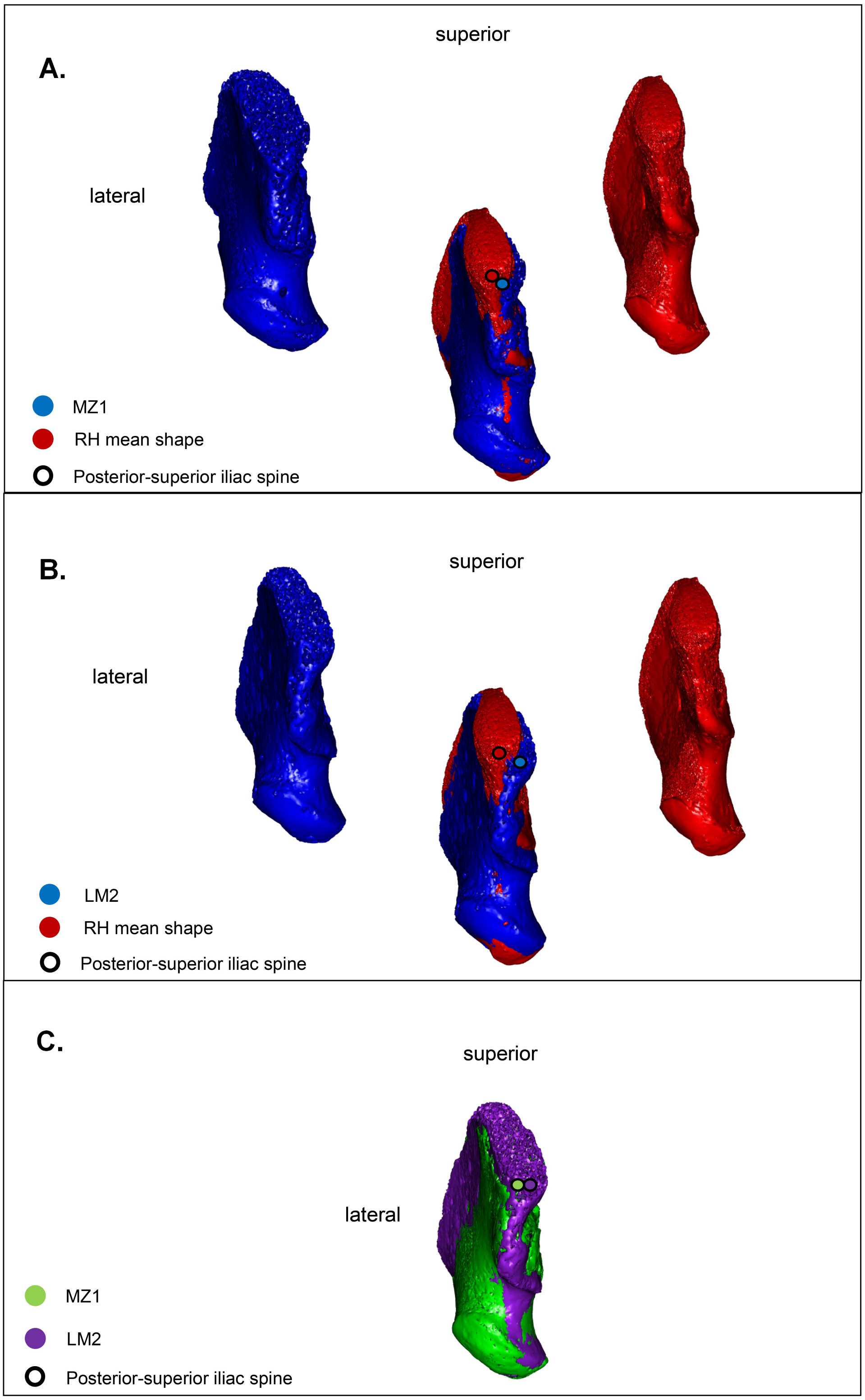

Supplementary Figure S5. A) Comparison between the ilium's shape of Mezmaiskaya 1 (MZ1, blue) and the mean shape of the recent perinatal individuals (RH, red) in posterior view. B) Same comparison between the perinatal Neanderthal Le Moustier (LM2, blue) and the mean shape of the recent perinatal individuals (RH, red). C) Same comparison between the perinatal Neanderthal Mezmaiskaya 1 (MZ1, green) and Le Moustier 2 (LM2, purple).

The figure was generated using R software (version 4.1.2) http://www.R-project.org/ and Adobe Illustrator (version 4.1.2) https://www.adobe.com/products/illustrator.html.
